# Dual sensory impairment: Global prevalence, future projections, and its association with cognitive decline

**DOI:** 10.1002/alz.14465

**Published:** 2025-01-30

**Authors:** Brian Sheng Yep Yeo, Esther Yanxin Gao, Benjamin Kye Jyn Tan, Benedict Ding Chao Ong, Ryan Wei Yang Cho, Chee Yit Lim, Ryan Eyn Kidd Man, Eva K. Fenwick, Preeti Gupta, Christopher Li‐Hsian Chen, Samuel Teong Huang Chew, Neville Wei Yang Teo, Song Tar Toh, Jia Hui Ng, Vanessa Yee Jueen Tan, Ecosse L. Lamoureux

**Affiliations:** ^1^ Yong Loo Lin School of Medicine National University of Singapore Singapore Singapore; ^2^ Department of Otorhinolaryngology‐Head and Neck Surgery Singapore General Hospital Singapore Singapore; ^3^ Department of Otorhinolaryngology‐Head and Neck Surgery Sengkang General Hospital Singapore Singapore; ^4^ Surgery Academic Clinical Program Duke‐NUS Medical School Singapore Singapore; ^5^ Singapore Eye Research Institute, Singapore National Eye Centre Singapore Singapore; ^6^ Ophthalmology and Visual Sciences Academic Clinical Programme Duke‐NUS Medical School Singapore Singapore; ^7^ Health Services and System Research Department Duke‐NUS Medical School Singapore Singapore; ^8^ Memory Aging and Cognition Centre Department of Pharmacology Yong Loo Lin School of Medicine National University of Singapore Singapore Singapore; ^9^ Department of Psychological Medicine National University Hospital Singapore Singapore Singapore; ^10^ Department of Geriatric Medicine Changi General Hospital Singapore Singapore; ^11^ SingHealth Duke‐NUS Medicine Academic Clinical Programme Singapore Singapore; ^12^ Faculty of Medicine Dentistry and Health Sciences The University of Melbourne Melbourne Victoria Australia

**Keywords:** cognitive decline, cognitive impairment, dementia, dual sensory impairment, hearing impairment, public health, vision impairment

## Abstract

There is rising public health concern surrounding dual sensory impairment (DSI), or comorbid hearing and vision impairments. Its global prevalence and the magnitude of its association with cognitive decline (CD) is unclear. Three databases were searched for epidemiological studies examining DSI prevalence or its association with CD. Independent reviewers selected studies, extracted data, and evaluated bias. Random‐effects meta‐analyses were performed. Projections were estimated using United Nations data. The population attributable fraction of DSI‐associated CD was calculated. Among 43 studies with 5,246,796 participants, clinically assessed DSI prevalence was 5.50% (95% confidence interval [CI] = 2.88%–10.26%), with regional/ethnic/age variations. DSI prevalence is projected to increase by 27.2% from 2025 to 2050. Approximately 59.83% (95%CI = 41.03–76.12) of DSI patients had cognitive impairment. Baseline DSI was associated with incident CD (odds ratio [OR] = 1.72, 95%CI = 1.37–2.15). Globally, 3.81% (95%CI = 1.05–10.55) of incident CD may be attributed to DSI. DSI is globally prevalent, growing, and associated with CD, highlighting the need for better health policy and resource allocation.

**Highlights:**

- The global prevalence of DSI is 5.50%, with geographical, ethnical and age variations.
- The prevalence of DSI rises with age and is projected to increase by 27.2% by 2050.
- Approximately 60% of individuals with DSI may have measurable cognitive impairment.
- DSI was associated with a 72% greater longitudinal risk of incident CD.
- Globally, 3.81% of CD cases may be attributable to DSI.

## 1 INTRODUCTION

Both hearing and vision impairments (HI and VI) are highly prevalent and associated with cognitive decline (CD) in older adults.^1^, ^2^ Worldwide, the number of people living with dementia is expected to increase from 57.4 million in 2019 to 152.8 million by 2050,^3^ while 15.6% of adults aged 50 years or more are living with mild cognitive impairment.^4^ Early detection of high‐risk individuals is thus essential to robustly manage the growing burden of CD. The Lancet Commission suggests that HI and VI are some of the largest modifiable risk factors for dementia,^5^, ^6^ and their correction has been shown to slow CD in at‐risk individuals.^7^, ^8^, ^9^, ^10^

While the burden of each of these two sensory impairments has been well‐described,^11^, ^12^ the consequences of dual sensory impairment (DSI) or comorbid VI and HI are less well‐defined. To date, the global prevalence of DSI has not been systematically examined, and the magnitude of its association with CD is unclear.^13^, ^14^

With a rapidly aging global population, the public health burden of DSI and CD is expected to grow. A precise estimate of the current prevalence and future projections of DSI, as well as its association with CD, is thus essential for the development of effective health policies. This systematic review and meta‐analysis seek to establish the global prevalence of DSI and its attributable risk of CD.

## 2 METHODS

This study was registered on PROSPERO (CRD42023452143) and conducted in accordance with the Preferred Reporting Items for Systematic Reviews and Meta‐Analyses (PRISMA) guidelines (Table S1).^15^

### 2.1 Search strategy and study selection

We systematically searched PubMed, Embase, and Cochrane Library databases from their inception till November 22, 2023. The search strategy consisted of the following keywords and associated synonyms: DSI, prevalence, cognitive impairment, and dementia. The full search strategy is outlined in the Supporting Information.

### 2.2 Study selection

The titles and abstracts, followed by full texts, of all identified articles were screened by three independent reviewers (B.D.C.O., R.W.Y.C., and C.Y.L.), with disputes resolved through the consensus of a fourth independent author (B.S.Y.Y.). We included observational studies published as full‐length publications in peer‐reviewed journals, with a population of adults aged at least 18 years old, if they reported either: (I) DSI prevalence in a population‐based setting from a defined geographical region or (II) the association of DSI with any prevalent cognitive impairment and incident CD. Given that we expected greater heterogeneity in the analysis of DSI prevalence, we accepted only DSI measured using clinically assessed subjective tests, such as pure‐tone audiometry (PTA) for hearing and logMAR charts for vision, respectively. Separately, we accepted both self‐reported and clinically measured VI and HI for the association between DSI and CD, albeit subject to further subgroup stratification. Any prevalent cognitive impairment was defined as a composite outcome comprising either prevalent cognitive impairment (defined by a binary threshold on a test of general cognition) or prevalent dementia (as diagnosed by a physician, or in accordance with accepted criteria such as the Diagnostic and Statistical Manual of Mental Disorders, or based on diagnostic codes, for example, International Classification of Disease—ICD).^16^ Incident CD was defined as a composite outcome comprising either an incident decline in test scores of general cognition (e.g., Mini‐Mental State Examination [MMSE]), or incident cognitive impairment, or incident dementia (same criteria as above).

We excluded studies based on the following exclusion criteria: (1) case reports, (2) reviews, (3) meta‐analyses, (4) letters, (5) conference abstracts and proceedings, (6) studies published in any language other than English, (7) pediatric studies, (8) animal studies, (9) studies which reported outcomes in special populations, and (10) reported the number of eyes or ears rather than the number of individuals.

### 2.3 Data extraction

The data extraction process was conducted by four independent reviewers (B.S.Y.Y., E.Y.G., B.D.C.O., and R.W.Y.C.). Thereafter, a separate independent reviewer (B.K.J.T.) verified the details of the extraction. We extracted pertinent data related to the characteristics of studies and the outcomes of interest into a structured template from shortlisted studies. Study characteristics of the included studies are outlined in Tables S2–S3.

### 2.4 Risk of bias assessment

Two independent reviewers (B.D.C.O. and R.W.Y.C.) assessed the risk of bias of observational studies using the Risk of Bias In Non‐Randomized Studies of Exposures (ROBINS‐E) tool, with disagreements resolved by two independent authors (B.S.Y.Y. and B.K.J.T.). The ROBINS‐E tool assesses the risk of bias in seven methodological domains, where bias in each domain was graded as either low, some, high, or very high concerns. The overall risk of bias for each study was determined using the highest risk of bias for any domain.

**RESEARCH IN CONTEXT**

1. **Systematic review**: The authors reviewed literature from PubMed, Embase, and Cochrane Library. The current and future global prevalence of dual sensory impairment (DSI) have not been systematically examined, and the association between DSI and cognitive decline (CD) is inconclusive.
2. **Interpretation**: The estimated current global prevalence of DSI is 5.50% and is projected to rise by 27.2% from 2025 to 2050. Crucially, 60% of individuals with DSI may have measurable cognitive impairment. DSI is also associated with a greater risk of CD. Globally, 3.81% of CD may be attributed to DSI. Cumulatively, our findings emphasize the importance of early detection and integrated management of hearing and vision impairments to mitigate their combined impact on cognition.
3. **Future directions**: The evidence underscores the urgent need for public health policies and healthcare practices to address DSI in aging populations, through proactive screening and community advocacy. Future randomized controlled trials may strive to elucidate the cognitive benefits of treating DSI and develop cost‐effective interventions.

### 2.5 Statistical analysis

The global prevalence of DSI was pooled using random‐effects meta‐analysis to allow for anticipated clinical heterogeneity. Whenever possible, generalized linear mixed models (GLMM) with logit‐transformed data were used to avoid errors associated with the double‐arcsine transformation. Between‐study heterogeneity was evaluated with the *I* ^2^ statistic (the percentage of variability due to heterogeneity rather than chance) and the Cochran Q test (where a *p*‐value of ≤0.10 was considered significant for heterogeneity).^17^, ^18^ As the *I* ^2^ value is well‐known to artificially inflate with increasing sample size despite constant heterogeneity,^17^ τ^2^ was concurrently estimated to directly quantify heterogeneity.

Patient‐level pre‐specified subgroup meta‐analyses stratified by geographical continent, ethnicity, and age (by decade) were conducted to estimate epidemiological variations in prevalence. We evaluated potential sources of heterogeneity for the pre‐specified variables of age, percentage male, study design, ethnicity, and continent via: (1) pre‐specified univariate meta‐regression analyses with inverse variance weights and thousand‐fold permutation testing if at least ten studies were present for continuous, binary or ordinal variables; (2) Q test was performed for heterogeneity across subgroups for multinomial variables.

Leave‐one‐out influence analyses and cumulative meta‐analyses were respectively used to evaluate the impact of individual studies on the pooled effect and to ascertain the stability of published data over time. Publication bias, which can be inferred from funnel plot asymmetry, was assessed qualitatively using visual inspection and quantitatively using Egger's test. Where publication bias was suspected, the trim‐and‐fill method was used to impute potentially missing studies and re‐estimate the pooled effect.

Global population projections of DSI were estimated using data from the United Nations World Population Prospects, which compile the most recent findings from national population census and demographic surveys worldwide, after accounting for mortality and fertility rates. DSI projections were calculated by multiplying categorical age‐specific and region‐specific pooled prevalence rates from the above meta‐analyses with the corresponding population distributions at 5‐year intervals up to 2050.

Separately, random‐effects inverse variance meta‐analysis was used to pool the maximally covariate‐adjusted association of DSI with prevalent and incident CD. Subgroup analyses were conducted using the following pre‐specified variables: the method of hearing and vision assessment (subjective vs. objective), type of cognitive outcome (decline in cognitive test scores vs. cognitive impairment vs. dementia), and comparator group (participants without DSI vs. participants with normal sensory function). Heterogeneity, meta‐regression, publication bias, influence, and cumulative meta‐analyses were subsequently assessed or conducted using the same methodology described above.

The age‐specific and region‐specific population attributable fraction (PAF) of incident CD associated with DSI was then calculated using the Miettinen formula: $PAF\%=\frac{p\times (RR-1)}{p\times (RR-1)+1}\;\times 100\%$, where *p* represents the prevalence of DSI in a given region, and *RR* represents the pooled relative risk of incident CD associated with DSI. Both values are derived from the above meta‐analyses. The PAF estimates the percentage of incident CD cases explained by DSI, assuming the absence of confounders. This analysis was repeated for incident dementia.

All statistical analyses were performed in R (4.0.3) using the following packages (version number): *meta* (4.18.1)*, metafor* (2.4.0), *dmetar* (0.0.9), *dplyr* (1.0.7), *ggplot2* (3.3.5), *scales* (1.1.1), and *pracma* (2.4.4). Unless otherwise specified, a two‐sided *p*‐value of ≤0.05 was considered statistically significant.

### 2.6 Overall quality of evidence

The overall quality of evidence per outcome was evaluated using the Grading of Recommendations, Assessment, Development, and Evaluations (GRADE) framework.^19^, ^20^ The GRADE framework assesses each outcome based on consistency, directness, risk of bias, precision, and publication bias. The level of evidence for each outcome may be categorized as high, moderate, low, or very low.

## 3 RESULTS

The initial search across databases identified 3009 results, of which 175 duplicates were deleted. The titles and abstracts of 2834 articles were screened, and 151 full‐text articles were examined for eligibility, with reasons for exclusion indicated in Figure S1. We included 43 studies and 5,246,796 participants in the quantitative synthesis.

### 3.1 Study characteristics

Approximately 41.8% of participants were male, and the mean age was 64.4 ± 14.9 years old. Fifteen studies were conducted in North America,^13^, ^21^, ^22^, ^23^, ^24^, ^25^, ^26^, ^27^, ^28^, ^29^, ^30^, ^31^, ^32^, ^33^ 14 in Asia,^34^, ^35^, ^36^, ^37^, ^38^, ^39^, ^40^, ^41^, ^42^, ^43^, ^44^, ^45^, ^46^, ^47^, ^48^ 10 in Europe,^49^, ^50^, ^51^, ^52^, ^53^, ^54^, ^55^, ^56^, ^57^, ^58^ 3 in Oceania,^14^, ^59^, ^60^ and 1 in both North America and Europe.^61^ All studies were observational, of which 16 and 27 were cross‐sectional,^32^, ^33^, ^35^, ^36^, ^39^, ^40^, ^41^, ^42^, ^43^, ^44^, ^47^, ^53^, ^55^, ^58^, ^59^, ^61^, and longitudinal^13^, ^14^, ^21^, ^22^, ^23^, ^24^, ^25^, ^26^, ^27^, ^28^, ^29^, ^30^, ^31^, ^34^, ^37^, ^38^, ^45^, ^46^, ^47^, ^48^, ^49^, ^50^, ^51^, ^52^, ^54^, ^56^, ^60^, respectively. For the global prevalence analysis, 15 studies had a low risk of bias,^25^, ^26^, ^31^, ^32^, ^33^, ^35^, ^38^, ^39^, ^41^, ^45^, ^46^, ^51^, ^53^, ^54^, ^59^ 8 with moderate risk of bias,^21^, ^22^, ^36^, ^42^, ^44^, ^49^, ^55^, ^60^ and no study had a high risk of bias. Separately, for studies included in the analyses between DSI and CD, 1 study had a low risk of bias,^14^ 14 had a moderate risk of bias,^23^, ^27^, ^29^, ^30^, ^37^, ^42^, ^46^, ^47^, ^48^, ^50^, ^56^, ^57^, ^58^, ^61^ and 8 had a high risk of bias (Tables S4–S5).^13^, ^24^, ^28^, ^34^, ^40^, ^43^, ^45^, ^52^

### 3.2 Global prevalence & projections of DSI

#### 3.2.1 Overall prevalence

**FIGURE 1:**
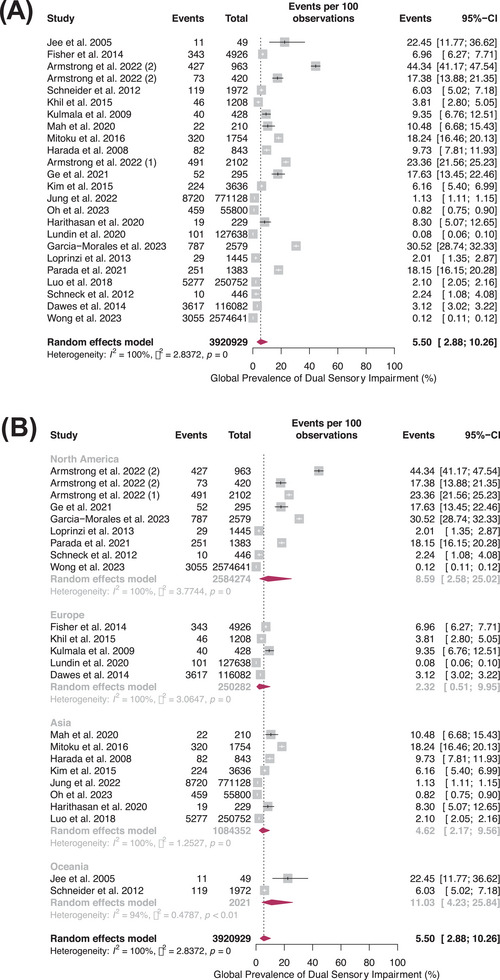
(A) Forest plot showing the pooled global prevalence of dual sensory impairment among adults aged at least 20 years. The maroon diamonds are the estimated pooled prevalence for each random effects generalized linear mixed model meta‐analysis; horizontal lines represent the 95% confidence intervals. (B) Forest plot showing the pooled global prevalence of dual sensory impairment among adults aged at least 20 years, stratified by continent at the study‐level. The maroon diamonds are the estimated pooled prevalence for each random effects generalized linear mixed model meta‐analysis; horizontal lines represent the 95% confidence intervals. (C) Forest plot showing the pooled global prevalence of dual sensory impairment among adults aged at least 20 years, stratified by ethnicity. The maroon diamonds are the estimated pooled prevalence for each random effects generalized linear mixed model meta‐analysis; horizontal lines represent the 95% confidence intervals.

Twenty‐three studies investigated the prevalence of DSI. Hearing and vision were measured using clinically assessed subjective tests, such as PTA, Snellen Charts, and Bailey‐Lovie Charts.^17^, ^18^, ^21^, ^22^, ^27^, ^28^, ^29^, ^31^, ^32^, ^34^, ^35^, ^37^, ^38^, ^40^, ^41^, ^42^, ^45^, ^47^, ^49^, ^50^, ^51^, ^55^, ^56^ Meta‐analysis estimated the pooled global prevalence of DSI at 5.50% (95% confidence interval (95%CI): 2.88–10.26%) (Figure 1A),^17^, ^18^, ^21^, ^22^, ^27^, ^28^, ^29^, ^31^, ^32^, ^34^, ^35^, ^37^, ^38^, ^40^, ^41^, ^42^, ^45^, ^47^, ^49^, ^50^, ^51^, ^55^, ^56^ with considerable between‐study heterogeneity (*I* ^2^ = 100%, τ^2^ = 2.88). Additional leave‐out‐one influence analysis (Figure S2) showed that no single study drastically changed the pooled effect size, while cumulative meta‐analysis (Figure S3) showed a stable pooled effect size since 2012. There was no funnel plot asymmetry detected by visual inspection or Egger's test (intercept = 22.01; *p* = 0.06) and, thus, no evidence of publication bias (Figure S4 and Table S6). The percentage of males and study design were not significant effect moderators (Table S7).

#### 3.2.2 Continent‐stratified prevalence

The pooled prevalence of DSI in each continent (Figure 1B) was as follows: North America (8.59%, 95% CI = 2.58%–25.02%, *I* ^2^ = 100%, τ^2^ = 3.77, eight studies); Europe (2.32%, 95% CI = 0.51%–9.95%, *I* ^2^ = 100%, τ^2^ = 3.06, five studies); Asia (4.62%, 95% CI = 2.17%–9.56%, *I* ^2^ = 100%, τ^2^ = 1.25, eight studies); and Oceania (11.03%, 95% CI = 4.23–25.84, *I* ^2^ = 94%, τ^2^ = 0.48, two studies). There was no significant difference between subgroups (*p* = 0.260), and data were unavailable for Africa.

#### 3.2.3 Ethnicity‐stratified prevalence

The pooled prevalence of DSI by ancestral ethnicity (Figure 1C) was as follows: European (5.74%, 95% CI = 2.17%–14.34%, *I* ^2^ = 100%, τ^2^ = 3.97, 15 studies); African (9.81%, 95% CI = 1.61%–41.87%, *I* ^2^ = 100%, τ^2^ = 4.63, 15 studies); and Asian (3.10%, 95% CI = 1.12%–8.26%, *I* ^2^ = 100%, τ^2^ = 2.50, 9 studies), with no significant difference between subgroups (*p* = 0.470).

#### 3.2.4 Age‐stratified prevalence

**FIGURE 2:**
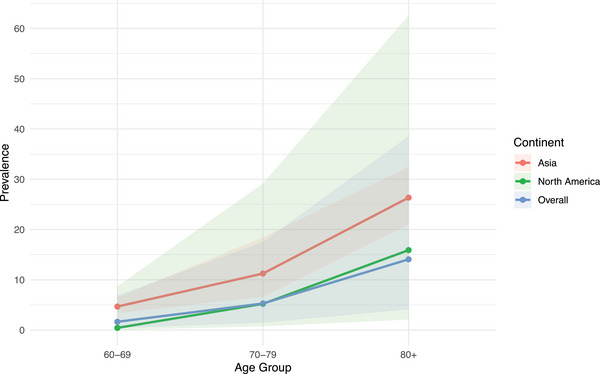
Line graph of pooled prevalence of dual sensory impairment, stratified by age and continent at the patient‐level. The dots represent the pooled mean prevalence; shaded bands represent the 95% confidence intervals of the respective means. Insufficient patient‐level data were available or for age groups below 60 years old and for the other continents of Europe, Oceania, and Africa.

The pooled global prevalence of DSI increased with age among older adults (Figure 2): 60–69 years old (1.65%, 95% CI = 0.38%–6.87%, *I* ^2^ = 100%, τ^2^ = 5.58, 10 studies) (Figure S5A); 70–79 years old (5.29%, 95% CI = 1.45%–17.53%, *I* ^2^ = 100%, τ^2^ = 5.45, 12 studies) (Figure S5B); ≥80 years old (14.07%, 95% CI = 4.09%–38.59%, *I* ^2^ = 100%, τ^2^ = 4.67, 12 studies) (Figure S5C). This association was verified on meta‐regression, where categorical age was a significant effect moderator (*p* = 0.029, Figure S6). The age‐dependent trend was also seen in continent‐specific analyses for Asia (4.67%; 11.24%; 25.00%) and North America (0.43%; 5.22%; 15.90%) for the respective age categories. Insufficient patient‐level data were available for younger adults or other continents.

#### 3.2.5 Global projections of DSI

**FIGURE 3:**
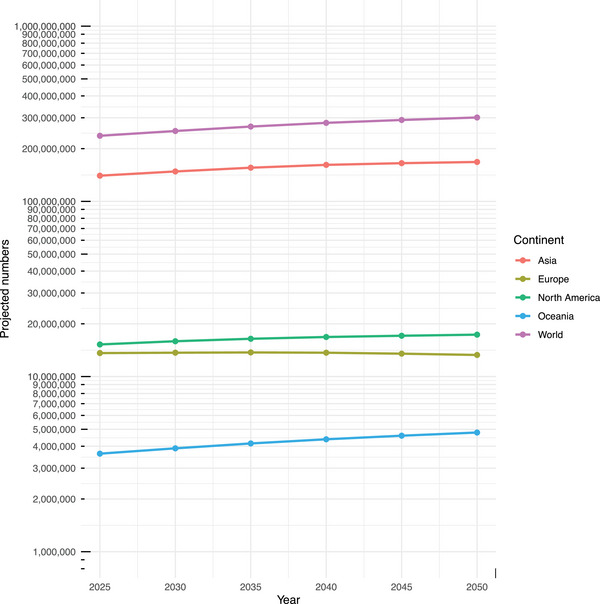
Line graph of future projections of dual sensory impairment, stratified by continent. The y‐axis is displayed on log_10_ scale. The dots represent the projected total number of people living with dual sensory impairment in each region, derived based on median region‐stratified projections from the United Nations World Population Prospects 2022. Projections for Africa are unavailable due to the absence of prevalence data from Africa.

The global population living with DSI is projected to increase from 236,850,106 persons in 2025 to 301,183,667 persons in 2050, an increase of 27.2% (Figure 3). The respective projections for each continent from 2025 to 2050 are: Asia (140,167,363 to 167,868,608, an increase of 19.8%), Europe (13,616,781 to 13,282,580, a decrease of 2.5%), North America (15,249,800 to 17,343,279, an increase of 13.7%), and Oceania (3,629,440 to 4,791,132, an increase of 32.0%). Data were unavailable for Africa.

### 3.3 Association of DSI with CD

#### 3.3.1 Assessment of DSI

Among the 23 studies investigating the association between DSI and cognition, vision was measured by clinically assessed subjective tests (e.g., Snellen Charts) in eight studies,^14^, ^23^, ^29^, ^42^, ^46^, ^50^, ^52^, ^58^ validated self‐report in ten studies (i.e., questionnaires, confirmed with ICD codes),^24^, ^27^, ^30^, ^37^, ^45^, ^47^, ^48^, ^56^, ^57^, ^61^ and unvalidated self‐report in five studies.^13^, ^28^, ^34^, ^40^, ^43^ Separately, hearing was evaluated using clinically assessed subjective tests (i.e., PTA) in six studies,^14^, ^23^, ^29^, ^42^, ^46^, ^52^ validated self‐report (e.g., Hearing Handicap Inventory for The Elderly Screening Version [HHIE‐S]) in 11 studies,^24^, ^27^, ^30^, ^37^, ^45^, ^56^, ^58^, ^61^ and unvalidated self‐report in 5 studies.^13^, ^28^, ^34^, ^40^, ^43^ Dintica and colleagues accepted both self‐reported and clinical assessments of HI identified via ICD codes.^50^

#### 3.3.2 Assessment of cognition

Among 23 studies, 17 studies investigated the outcome of cognitive impairment,^13^, ^23^, ^24^, ^27^, ^31^, ^34^, ^36^, ^37^, ^40^, ^43^, ^44^, ^45^, ^47^, ^51^, ^57^, ^58^, ^61^ as measured by validated means of cognitive testing such as MMSE; 9 studies examined the outcome of dementia,^28^, ^30^, ^34^, ^42^, ^46^, ^48^, ^50^, ^52^, ^56^ as defined by validated means such as DSM criteria; 2 studies investigated the outcome of decline in cognitive test scores.^14^, ^29^ The full list of tools used to assess cognition is outlined in Table S2.

#### 3.3.3 Cross‐sectional association of DSI with any prevalent cognitive impairment

Among patients with DSI, the pooled prevalence of cognitive impairment and dementia was 59.83% (95% CI = 41.03–76.12, *I* ^2^ = 99%, τ^2^ = 0.73, five studies) and 5.20% (95% CI = 1.63–15.34, *I* ^2^ = 100%, τ^2^ = 1.11, three studies), respectively (Figure S7).

**FIGURE 4:**
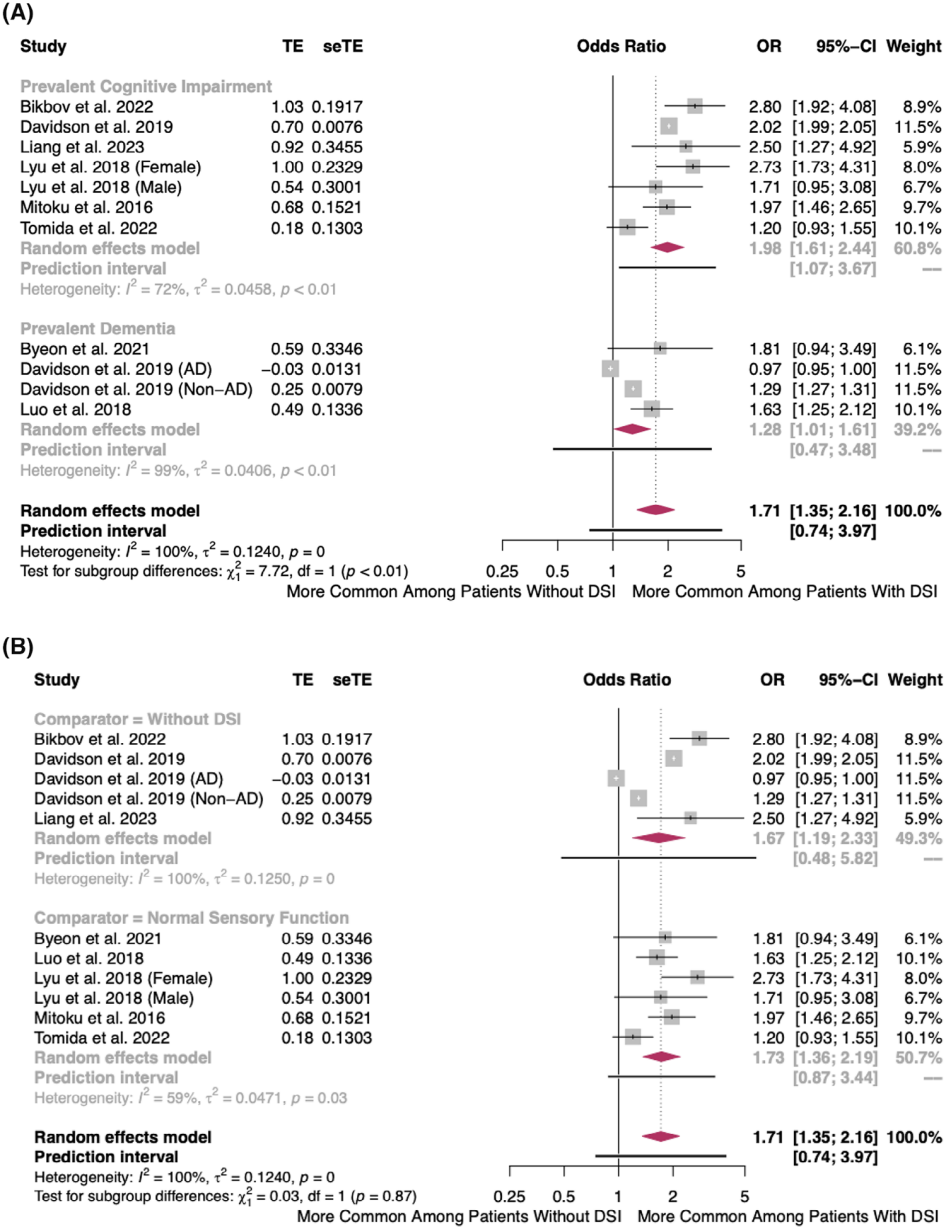
(A) Forest plot showing the cross‐sectional association between dual sensory impairment and prevalent cognitive impairment, stratified by cognitive outcome. The maroon diamonds are the estimated pooled odds ratio for each random‐effects meta‐analysis; gray box sizes reflect the relative weight apportioned to studies in the meta‐analysis; horizontal lines represent the 95% confidence and prediction intervals, respectively. (B) Forest plot showing the cross‐sectional association between dual sensory impairment and prevalent cognitive impairment, stratified by comparator. The maroon diamonds are the estimated pooled odds ratio for each random‐effects meta‐analysis; gray box sizes reflect the relative weight apportioned to studies in the meta‐analysis; horizontal lines represent the 95% confidence and prediction intervals, respectively.

DSI was associated with 71% greater cross‐sectional odds of any prevalent cognitive impairment (OR = 1.71, 95% CI = 1.35–2.16, 95% prediction interval [PI] = 0.74–3.97, *I* ^2^ = 100%, τ^2^ = 0.12, eight studies) (Figure 4A). When stratified by the type of cognitive outcome, DSI was associated with 98% and 28% greater cross‐sectional odds of cognitive impairment (OR = 1.98, 95% CI = 1.61–2.44, 95% PI = 1.07–3.67, *I* ^2^ = 72%, τ^2^ = 0.05, seven studies) and dementia (OR = 1.28, 95%CI = 1.01–1.61, 95%PI = 0.47–3.48, *I* ^2^ = 99%, τ^2^ = 0.04, four studies), respectively. All except one study measured DSI using self‐report (Figure 4A). The association was similar regardless of whether the comparison group included patients with single sensory impairment (Figure 4B). Further sensitivity analyses demonstrated consistent and stable findings (Figures S8–S9). There was no visual or quantitative funnel plot asymmetry to suggest publication bias (Figure S10, Egger's test Intercept = −0.89, *p* = 0.90).

#### 3.3.4 Longitudinal association of DSI with incident CD

**FIGURE 5:**
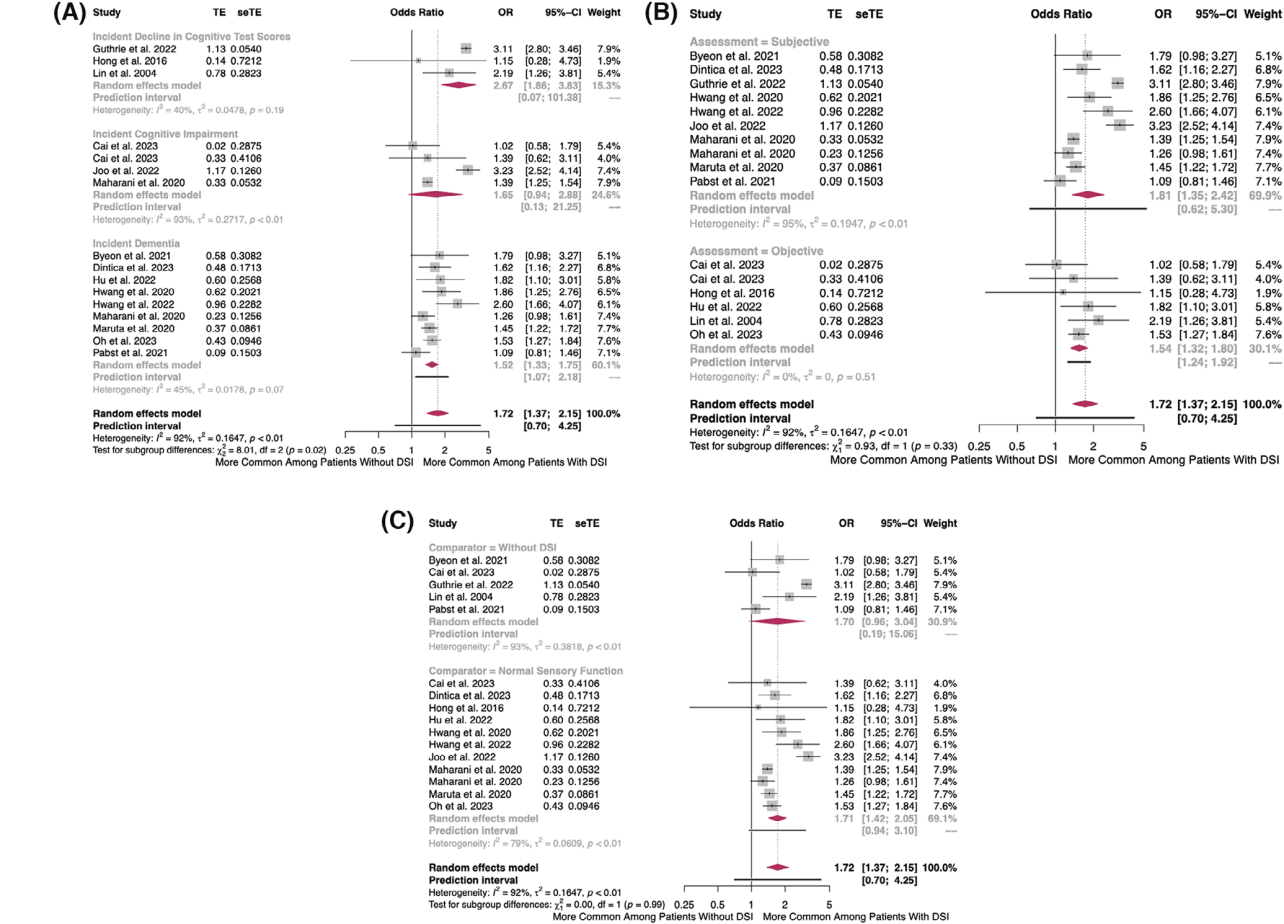
(A) Forest plot showing the longitudinal association between baseline dual sensory impairment and incident cognitive impairment, stratified by cognitive outcome. The maroon diamonds are the estimated pooled odds ratio for each random‐effects meta‐analysis; gray box sizes reflect the relative weight apportioned to studies in the meta‐analysis; horizontal lines represent the 95% confidence and prediction intervals, respectively. (B) Forest plot showing the longitudinal association between baseline dual sensory impairment and incident cognitive impairment, stratified by method of sensory ascertainment. The maroon diamonds are the estimated pooled odds ratio for each random‐effects meta‐analysis; gray box sizes reflect the relative weight apportioned to studies in the meta‐analysis; horizontal lines represent the 95% confidence and prediction intervals, respectively. (C) Forest plot showing the longitudinal association between baseline dual sensory impairment and incident cognitive impairment, stratified by comparator. The maroon diamonds are the estimated pooled odds ratio for each random‐effects meta‐analysis; gray box sizes reflect the relative weight apportioned to studies in the meta‐analysis; horizontal lines represent the 95% confidence and prediction intervals, respectively.

Baseline DSI was associated with 72% greater odds of incident CD (OR = 1.72, 95%CI = 1.37–2.15, 95%PI = 0.70–4.25, *I* ^2^ = 92%, τ^2^ = 0.16, 16 studies) (Figure 5A), with a consistent trend among the subgroups of incident decline in cognitive test scores (OR = 2.67, 95%CI = 1.86–3.83, 95%PI = 0.07–101.38, *I* ^2^ = 40%, τ^2^ = 0.05, 3 studies), incident cognitive impairment (OR = 1.65, 95%CI = 0.94–2.88, 95%PI = 0.13–21.25, *I* ^2^ = 93%, τ^2^ = 0.27, 4 studies), and incident dementia (OR = 1.52, 95%CI = 1.33–1.75, 95%PI = 1.07–2.18, *I* ^2^ = 45%, τ^2^ = 0.02, 9 studies) (Figure 5A). The trend was also similar regardless of whether the comparison group included patients with single sensory impairment (Figure 5C).

Self‐reported DSI was a key contributor to clinical and statistical heterogeneity, as heterogeneity was non‐existent among studies that measured DSI using clinically assessed subjective tests (OR = 1.54, 95%CI = 1.32–1.80, 95%PI = 1.24–1.92, *I* ^2^ = 0%, τ^2^ = 0.00, 6 studies) but was expectedly high among studies that measured DSI via self‐report (OR = 1.81, 95%CI = 1.35–2.42, 95%PI = 0.62–5.30, *I* ^2^ = 95%, τ^2^ = 0.19, 10 studies) (Figure 5B).

Age, study design, follow‐up duration, and DSI assessment were not significant effect moderators on meta‐regression (Table S7). Further sensitivity analyses demonstrated consistent and stable findings (Figures S11–S12). There was no visual or quantitative funnel plot asymmetry to suggest publication bias (Figure S13, Egger's test Intercept = −1.01, *p* = 0.52).

### 3.4 PAF of incident CD and dementia associated with DSI

The global PAF of CD and dementia associated with DSI was estimated at 3.81% (95%CI = 1.05–10.55) and 2.78% (95%CI = 0.94–7.15), respectively. This increased with age for both CD and dementia, respectively: 1.17% (95%CI = 0.14%–7.32%) and 0.85% (95%CI = 0.12–4.90) at age 60–69 years; 3.67% (95%CI = 0.53%–16.78%) and 2.68% (95%CI = 0.47–11.62) at age 70–79 years; 9.20% (95%CI = 1.49%–30.74%) and 6.82% (95%CI = 1.33%–22.45%) at age ≥80 years. The continent‐ and age‐specific PAFs are reported in Tables S8 and S9, with the highest PAF of 15.94% (95%CI = 7.22%–27.18%) for CD and 12.05% (95%CI = 6.49–19.58) for dementia in older Asian participants aged ≥80 years.

### 3.5 Quality of evidence

The overall quality of evidence was moderate (Table S10).

## 4 DISCUSSION

This meta‐analysis of 43 observational studies with 5,246,796 participants offers a comprehensive and contemporary estimate of the current and projected global prevalence of DSI, and its association with CD. The estimated current global prevalence of DSI is 5.50%, increasing up to 26% among Asians aged at least 80 years. DSI is common globally across all continents, and the number of people living with DSI is projected to grow by 27.2% over the next 25 years to 301,183,667 persons in 2050. DSI is also associated with prevalent and incident CD. We estimate that 3.81% and 16% of incident CD globally and among older Asians can be explained by DSI. Importantly, 60% of people living with DSI may also have measurable cognitive impairment.

The age‐dependent trends in DSI prevalence are consistent with previous prospective cohort studies.^25^, ^53^, ^62^ Age‐related sensory diseases, including presbycusis, cataracts, refractive error, age‐related macular degeneration, and glaucoma are expected to have greater public health ramifications.^11^, ^12^ In fact, most cases of hearing and vision loss are attributable to these age‐related causes.^63^, ^64^ Crucially, we anticipate the prevalence of DSI, and its attributable risk of CD, to be the greatest in the oldest‐old, aged at least 80 years. These findings concur with other studies which have demonstrated an exponential rise in the prevalence of CD with age.^65^, ^66^ Even as the percentage prevalence of DSI rises with age, the absolute number of older adults is also burgeoning.^67^ In 2018, individuals aged at least 65 years outnumbered children under 5 years for the first time,^68^ and the number of older adults aged at least 65 years will double to two billion by 2050.^68^ The oldest‐old demographic are among the most rapidly growing age group globally, and they exhibit some of the highest rates of both morbidity and mortality. This presents a major public health challenge across various health and social care systems. Given the trend of increasing life expectancies across various economies,^69^ it is essential to be cognizant of the growing number of the world's aging population in the oldest age groups, and the attendant rise in DSI prevalence.

While we estimate a future overall increase in DSI, some regions are more likely to be affected than others. It is anticipated that Asia may experience the highest absolute number of DSI cases, owing to its status as the most populous continent and as the current older populace (above 60 years) matures and transitions to become the oldest old (above 80 years) by 2050. Although the percentage prevalence of DSI in Asia may be currently lower than in other continents, the magnitude of older people residing in Asia contributes substantially to the absolute number of cases in the coming decades. The greatest increase in absolute numbers of people with DSI is expected in Oceania and Asia. Conversely, North America and Europe may experience a more gradual increase in DSI cases, driven by slower population growth rates among older adults in these regions compared to Asia. Notably, the observed decline in DSI prevalence predicted in Europe from 2040 to 2050 may be attributed to anticipated population decline in Europe after 2040. Overall, these findings underscore the need to plan for future care services for sensory impairment.

Our study adds value to existing evidence on the association between VI, HI, and CD, by studying the cumulative effects of concurrent VI and HI. In our meta‐analysis, patients with DSI had a 72% greater longitudinal risk of CD. When compared to existing meta‐analyses investigating single sensory impairment, the risk of CD from DSI appears greater. Shang and colleagues suggested that those with VI had a 35% (RR: 1.35, 95%CI: 1.28–1.41) and 47% (RR: 1.47, 95%CI: 1.36–1.60) greater risk of cognitive impairment and dementia, respectively.^70^ Separately, Loughrey and associates outlined that patients with presbycusis had a 22% (OR: 1.22 95%CI: 1.09–1.36) and 28% (OR: 1.28, 95%CI: 1.02–1.59) greater odds of cognitive impairment and dementia, respectively.^1^ While we should be cautious when indirectly comparing our data to these findings since study variables may differ, our results may reasonably imply synergistic effects of concomitant VI and HI in exacerbating the risk of CD.

The possible synergistic risk of DSI on CD may be explained by several factors. Individually, sensory impairments carry a risk of CD, which may be mediated by communication difficulties, social isolation,^71^ poorer mental and functional health,^72^ and greater cognitive load to accommodate visual and auditory deficits.^73^ These factors have been extensively discussed elsewhere.^71^, ^72^, ^73^ However, we wish to highlight that patients with DSI face even greater cognitive challenges due to the loss of compensatory mechanisms. Individuals with single sensory loss can often compensate for their impairment by sharpening the multi‐sensory integration of their remaining senses.^74^ For instance, those with HI may rely more on visual speech reading (lip reading), while individuals with VI exhibit better auditory judgments. Conversely, individuals with DSI are unable to compensate in this manner. Therefore, it is clinically plausible that VI and HI do not act individually but instead act synergistically to worsen cognition.

It would also be equally important to recognize other barriers to treatment for sensory impairment. Existing societal stigma surrounding the treatment of sensory impairments, which may be more pronounced in some cultures, presents a significant barrier to encouraging individuals to seek care.^75^, ^76^ For instance, concerns about losing independence frequently deter individuals from adopting hearing restorative devices or pursuing vision‐restoring surgery.^77^ Furthermore, voluntary neglect of treatment may be more pronounced in regions where cultural norms or misconceptions about aging and disability influence individuals to delay or forgo necessary interventions. A recent study by Shakarchi and colleagues found that older adults in the United States with VI or HI encounter higher levels of everyday discrimination than those without sensory impairments, with individuals having DSI experiencing even greater levels of discrimination.^78^ Thus, it is essential to adopt a holistic approach in public health advocacy for hearing and vision health—one that not only encourages treatment but also promotes greater societal awareness about VI and HI.

The findings of this study complement other meta‐analyses, reinforcing the significant consequences of DSI on aging. For example, Tan and colleagues outlined that DSI was associated with a 40% greater risk of all‐cause mortality.^79^ Collectively, these findings serve as a catalyst call for physicians and policymakers to adopt a more cautionary stance to patients with multiple sensory impairments. Importantly, given that no cure exists for dementia, preventive health strategies remain paramount. Considering the potential negative effects of DSI on various facets of aging, community advocacy and proactive screening initiatives play a key role in identifying hearing and vision loss, which may empower early intervention to be taken.

Having established the association of DSI with cognition, the next question is whether this risk can be modified by interventions. While previous observational meta‐analyses have investigated whether the treatment of VI and HI may modify the risk of CD, we are not aware of studies examining the cognitive benefits of treating DSI.^8^, ^10^ Moreover, apart from the landmark ACHIEVE trial by Lin and colleagues,^7^ there is a lack of randomized controlled trials (RCTs) investigating the cognitive benefits of treating sensory impairment, partly due to the ethical complexities of withholding sight or hearing‐restoring treatment for the control group. As highlighted by The Lancet Global Health Commission on Global Eye Health, over 90% of cases of VI are preventable or correctable using existing cost‐effective treatments.^80^ Hence, RCTs are essential to understand the cognitive impact of correcting VI and HI, and whether the benefit is larger for patients with DSI.

This study strengths lie in its adherence to a pre‐defined protocol following international guidelines. We included studies from various settings, enhancing the generalizability of our findings. The global prevalence analysis focused on studies with community‐based participants and validated assessments of hearing and vision, and patient‐level analyses revealed age and ethnicity influences on prevalence. DSI projections highlighted potential trends with significant public health policy implications. The association between DSI and CD was examined using maximally‐adjusted estimates to account for confounders, with findings robust to subgroup and sensitivity analyses, and no evidence of publication bias.

Nevertheless, this study has several limitations. First, data were unavailable for Africa, and Oceania and Europe had insufficient studies with data amenable to categorization by age groups in older adults. Second, underrepresentation of low‐ and middle‐income countries with limited access to screening and diagnostic services in available data may result in an underestimation of DSI in Asia. Third, the exclusion of non‐English articles may have introduced selection bias. Fourth, age‐specific prevalences may change in the coming decades because of shifting population demographics globally. Fifth, we observed a high degree of between‐study heterogeneity, which may represent unmeasured clinical variations. Sixth, as an observational meta‐analysis, this study cannot draw causative conclusions due to residual confounding, though maximally adjusted estimates were pooled to account for various covariates and our findings remained robust across various subgroup and sensitivity analyses. Seventh, while clinical assessments of hearing and vision were required for the global prevalence analysis, the analysis on DSI and CD included self‐reported assessments of DSI, of which a higher degree of heterogeneity was observed in the subgroup comprising of articles that utilized self‐reported testing. Nevertheless, subgroup analyses, comparing self‐reported and validated measures, showed robust pooled effect sizes in both groups. Finally, the severity of hearing and vision loss was not accounted for. Patients with both severe‐to‐profound HI and VI may face difficulty comprehending tasks in cognitive tests. Cumulatively, these limitations highlight the importance of further investigation into this field of research.

## 5 CONCLUSION

This meta‐analysis and projection analysis comprising 43 observational studies and 5,246,796 participants provides comprehensive estimates that reflect the present and future global prevalence of DSI, and its association with CD. The estimated current global prevalence of DSI is 5.50%, increasing up to 26% among Asian octogenarians. DSI is projected to rise by 27.2% from 2025 to 2050. Crucially, approximately 60% of individuals with DSI may have measurable cognitive impairment. DSI is also associated with both greater cross‐sectional odds and longitudinal risk of CD, with a PAF of 3.81% globally, increasing to almost 16% in Asian octogenarians. This study underscores the urgent need to address sensory impairment and its impact on cognition from a public health standpoint, to guide resource allocation amidst a rapidly aging global population.

## AUTHOR CONTRIBUTIONS

B.S.Y.Y., E.Y.G., and B.K.J.T contributed equally. B.S.Y.Y., B.K.J.T., E.K.F., P.G., R.E.K.M., and E.L.L. conceived and designed the study. B.S.Y.Y., B.D.C.O., R.W.Y.C., and C.Y.L. selected the articles and extracted data. B.K.J.T., B.S.Y.Y., and E.Y.G. were responsible for statistical analysis. B.S.Y.Y., E.Y.G., and B.K.J.T. wrote the original draft of the manuscript. R.E.K.M., E.K.F., P.G., C.L.C., S.T.H.C., N.W.Y.T., S.T.T., J.H.N., V.Y.J.T., and E.L.L. provided supervision and advice at different stages. B.S.Y.Y., E.Y.G., B.K.J.T., B.D.C.O., R.W.Y.C., C.Y.L., R.E.K.M., E.K.F., P.G., C.L.C., S.T.H.C., N.W.Y.T., S.T.T., J.H.N., V.Y.J.T., and E.L.L. were involved in writing, reviewing, and editing the manuscript. All authors have read and approved the final version of the manuscript. E.L.L. is the corresponding author. The corresponding author attests that all listed authors meet authorship.

## CONFLICT OF INTEREST STATEMENT

The authors declare no conflicts of interest. All authors declare that no support was received from any organization for the submitted work, no financial relationships with any organizations were established that might have an interest in the submitted work in the previous 3 years, and no other relationships or activities could have influenced the submitted work. Author disclosures are available in the Supporting Information.

## Supporting information



Supporting Information

Supporting Information

## Data Availability

Data will be made available upon reasonable request to the corresponding author. Detailed data from individual studies are available in the individual published articles.
