# Supplementary material for: Dual sensory impairment: Global prevalence, future projections, and its association with cognitive decline
Source: Alzheimers Dement. 2025 Jan 30;21(2):e14465. doi: 10.1002/alz.14465 (PMC11851313; doi:10.1002/alz.14465)
Supplement: Supplementary file 1 — Supporting Information [file ALZ-21-e14465-s001.docx]

[SUPPLEMENTAL METHODS 2](#_Toc165130752)

[Free Text Search Strategy 2](#_Toc165130753)

[Supplemental Figure S1 3](#_Toc165130754)

[Supplemental Figure S2 4](#_Toc165130755)

[Supplemental Figure S3 5](#_Toc165130755)

[Supplemental Figure S4 6](#_Toc165130756)

[Supplemental Figure S5 7](#_Toc165130757)

[Supplemental Figure S6 10](#_Toc165130758)

[Supplemental Figure S7 11](#_Toc165130759)

[Supplemental Figure S8 12](#_Toc165130760)

[Supplemental Figure S9 13](#_Toc165130761)

[Supplemental Figure S10 14](#_Toc165130762)

[Supplemental Figure S11 15](#_Toc165130763)

[Supplemental Figure S12 16](#_Toc165130764)

[Supplemental Figure S13 17](#_Toc165130765)

[SUPPLEMENTAL TABLES 18](#_Toc165130767)

[Supplemental Table S1 18](#_Toc165130768)

[Supplemental Table S2 22](#_Toc165130769)

[Supplemental Table S3 27](#_Toc165130769)

[Supplemental Table S4 30](#_Toc165130770)

[Supplemental Table S5 31](#_Toc165130771)

[Supplemental Table S6 32](#_Toc165130772)

[Supplemental Table S7 33](#_Toc165130773)

[Supplemental Table S8 34](#_Toc165130774)

[Supplemental Table S9 34](#_Toc165130774)

[Supplemental Table S10 35](#_Toc165130774)

**SUPPLEMENTAL METHODS**

## Free Text Search Strategy

We searched PubMed, Embase and Cochrane Library using the following free text search strategy on 22 November 2023

| # | Search Term |
| --- | --- |
| 1 | ((“dual sensory” OR “dual-sensory” OR “multiple sensory” OR “multiple-sensory” OR (hearing AND vision)) AND (impairment OR loss OR decline OR deficit OR difficulty)) |
| 2 | (((cognitive OR cognition OR neurocognitive) AND (dysfunction OR deficit OR impairment OR decline OR defect OR function OR disorder OR disease)) OR alzheimer OR dementia) |
| 3 | (prevalence OR prevalent OR incidence OR incident) |
| 4 | **1 AND (2 OR 3)**  ((“dual sensory” OR “dual-sensory” OR “multiple sensory” OR “multiple-sensory” OR (hearing AND vision)) AND (impairment OR loss OR decline OR deficit OR difficulty)) AND ((((cognitive OR cognition OR neurocognitive) AND (dysfunction OR deficit OR impairment OR decline OR defect OR function OR disorder OR disease)) OR alzheimer OR dementia) OR (prevalence OR prevalent OR incidence OR incident)) |

#

#

**SUPPLEMENTAL FIGURES**

Supplemental Figure S1**: PRISMA Flow Chart.**


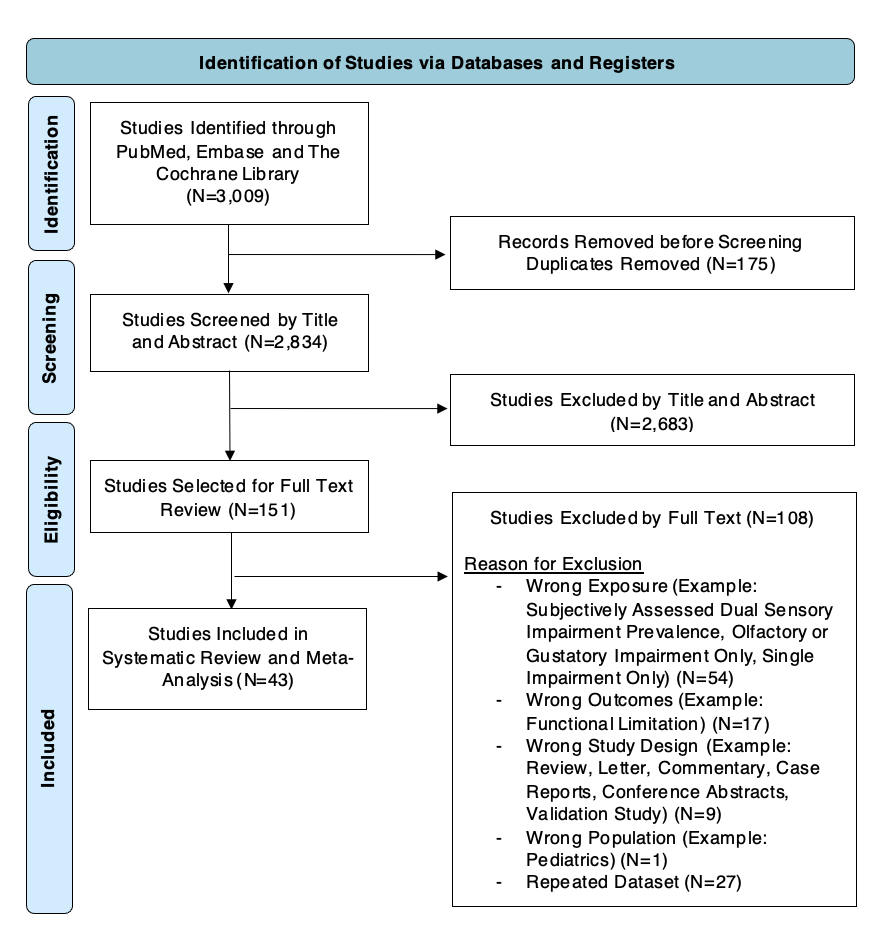


Supplemental Figure S2**: Leave-Out-One Influence Analysis of The Pooled Global Prevalence of Dual Sensory Impairment.**

Legend: The gray diamonds are the estimated pooled Hazard Ratio (HR) for each random-effects meta-analysis; gray box sizes reflect the relative weight apportioned to studies in the meta-analysis; proportions are displayed in this plot as a ratio.


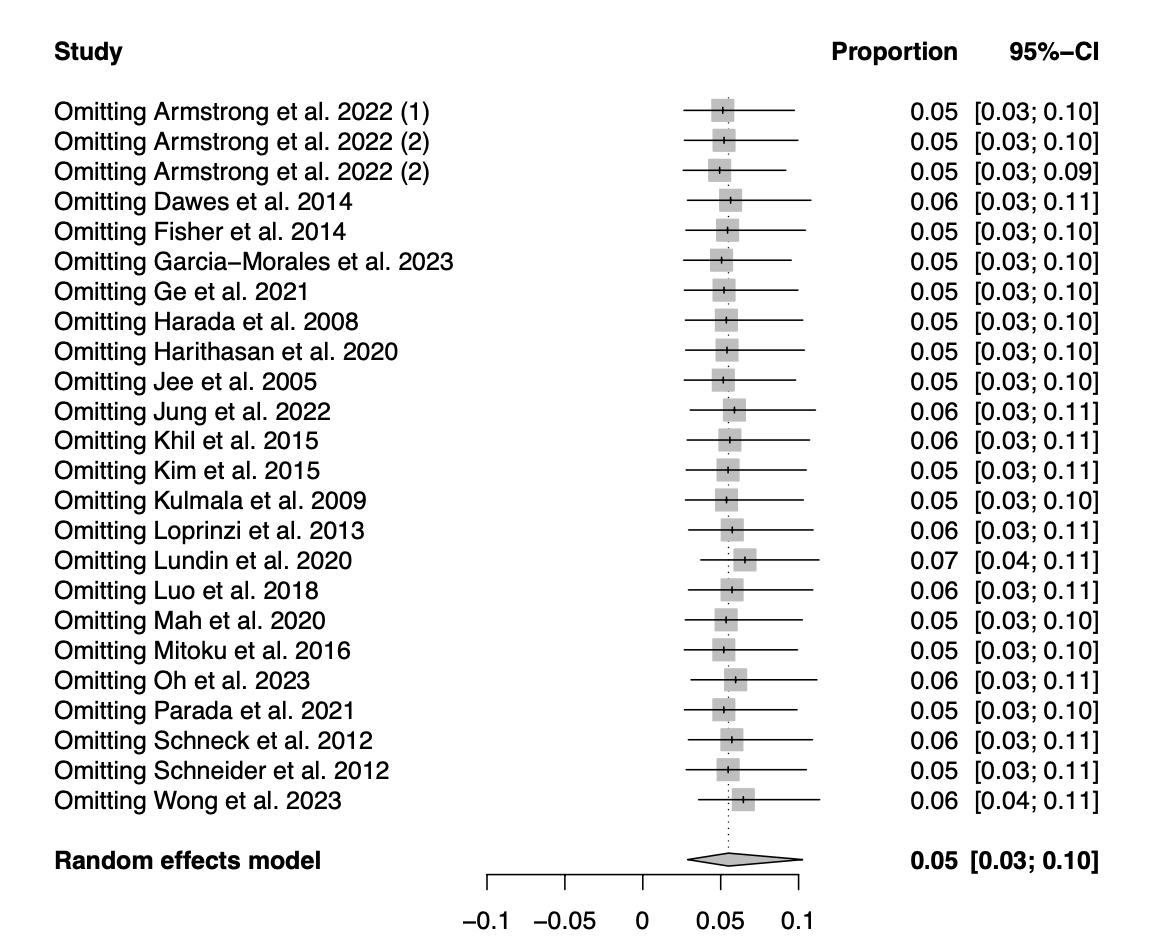


Supplemental Figure S3**: Cumulative Meta-Analysis, By Year Published, of The Pooled Global Prevalence of Dual Sensory Impairment.**

Legend: The gray diamonds are the estimated pooled Hazard Ratio (HR) for each random-effects meta-analysis; gray box sizes reflect the relative weight apportioned to studies in the meta-analysis; proportions are displayed in this plot as a ratio.


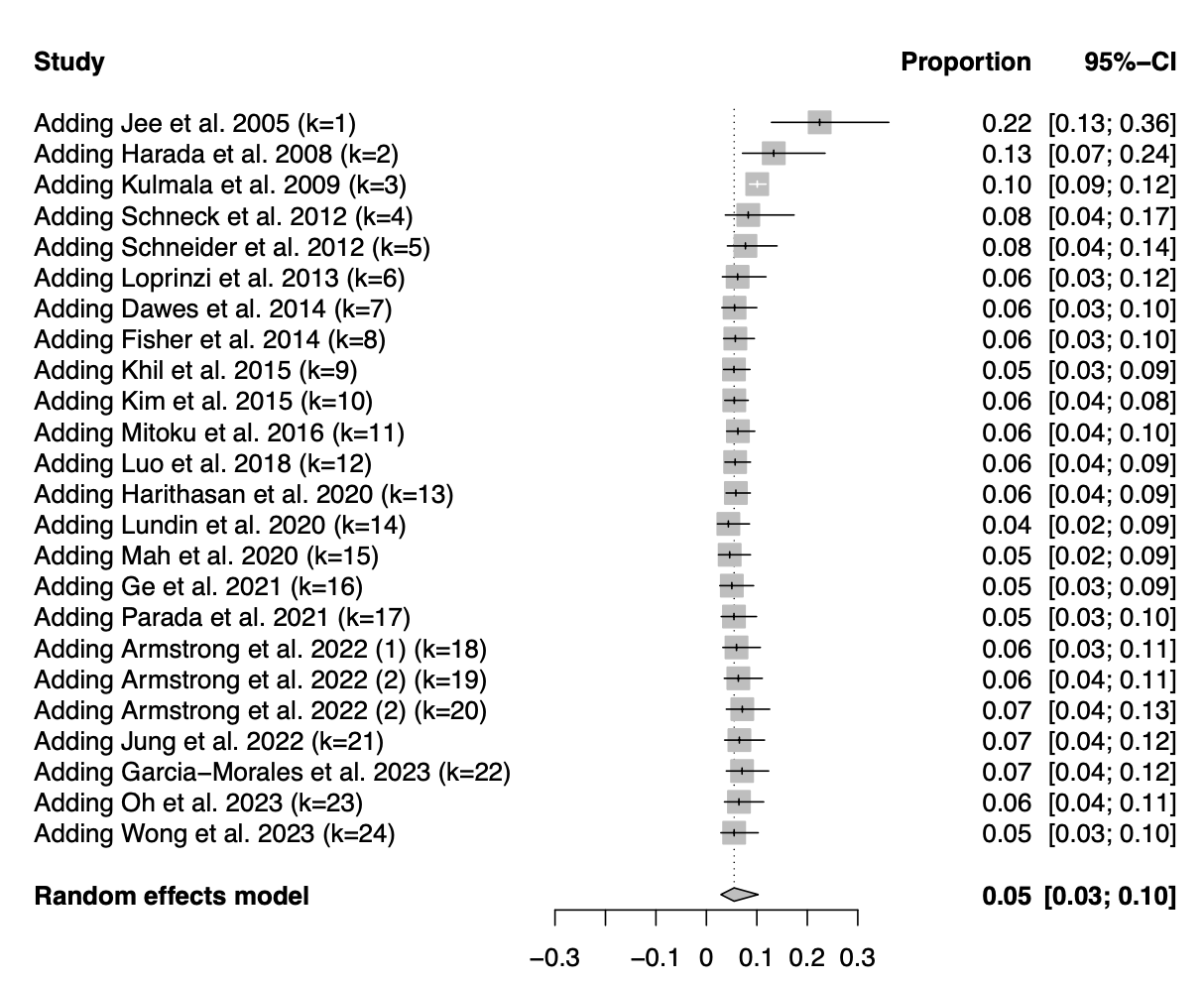


Supplemental Figure S4**: Contour-Enhanced Funnel Plot for Global Prevalence of Dual Sensory Impairment, With Missing Studies Imputed Using The Trim-And-Fill Method.**

Legend: The shaded circles represent the original study estimates and the unshaded circle represents the missing estimate imputed via the trim-and-fill method. The dark gray, gray and light gray contour lines indicate conventional milestones in levels of statistical significance (P<0.1, 0.05, 0.01).


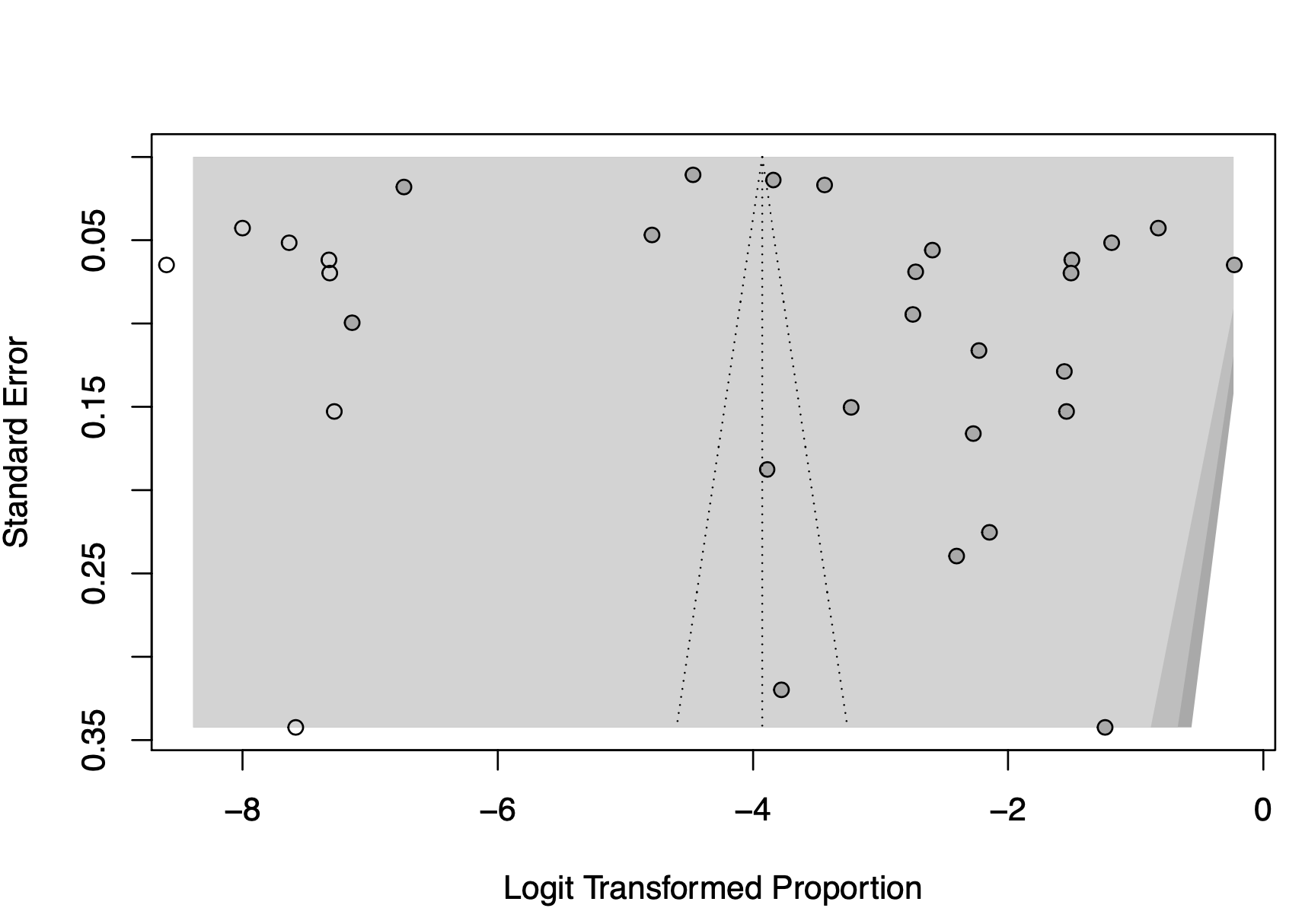


Supplemental Figure S5**: Forest Plot Showing The Pooled Prevalence of Dual Sensory Impairment Stratified by Continent, for Adults Aged (A) 60-69, (B) 70-79 and (C) ≥ 80 Years Old.**

Legend: The red diamonds are the estimated pooled prevalence for each random effects generalized linear mixed model meta-analysis; horizontal lines represent the 95% confidence intervals.

Supplementary Figure 5A: Prevalence of Dual Sensory Impairment Among Adults Aged 60-69 Years Old.

Supplementary Figure 5B: Prevalence of Dual Sensory Impairment Among Adults Aged 70-79 Years Old.

Supplementary Figure 5C: Prevalence of Dual Sensory Impairment Among Adults Aged ≥ 80 Years Old.

Supplemental Figure S6**: Bubble Plot for Random-Effects Meta-Regression of Logit Transformed Prevalence of Dual Sensory Impairment Against Age Group at The Patient-Level.**

Legend: The gray bubbles each represent one study and are plotted according to the study’s logit transformed proportion and age group; bubble sizes reflect the relative weight apportioned to studies in the random-effects meta-regression; the line of best fit is indicated in blue.

Supplemental Figure S7**: Forest Plot Showing The Pooled Prevalence of Cognitive Impairment and Dementia, Among Patients With Dual Sensory Impairment.**

Legend: The red diamonds are the estimated pooled prevalence for each random effects generalized linear mixed model meta-analysis; horizontal lines represent the 95% confidence intervals.

Supplemental Figure S8**: Leave-Out-One Influence Analysis of The Cross-Sectional Association of Dual Sensory Impairment With Prevalent Cognitive Impairment.**

Legend: The gray diamonds are the estimated pooled Hazard Ratio (HR) for each random-effects meta-analysis; gray box sizes reflect the relative weight apportioned to studies in the meta-analysis.

Supplemental Figure S9**: Cumulative Meta-Analysis, By Year Published, of The Cross-Sectional Association of Dual Sensory Impairment With Prevalent Cognitive Impairment.**

Legend: The gray diamonds are the estimated pooled Hazard Ratio (HR) for each random-effects meta-analysis; gray box sizes reflect the relative weight apportioned to studies in the meta-analysis.

Supplemental Figure S10**: Contour-Enhanced Funnel Plot for The Cross-Sectional Association of Dual Sensory Impairment With Prevalent Cognitive Impairment, With Missing Studies Imputed Using The Trim-And-Fill Method.**

Legend: The shaded circles represent the original study estimates and the unshaded circle represents the missing estimate imputed via the trim-and-fill method. The dark gray, gray and light gray contour lines indicate conventional milestones in levels of statistical significance (P<0.1, 0.05, 0.01).

Supplemental Figure S11**: Leave-Out-One Influence Analysis of The Longitudinal Association of Baseline Dual Sensory Impairment With Incident Cognitive Decline.**

Legend: The gray diamonds are the estimated pooled Hazard Ratio (HR) for each random-effects meta-analysis; gray box sizes reflect the relative weight apportioned to studies in the meta-analysis.

Supplemental Figure S12**: Cumulative Meta-Analysis, By Year Published, of The Longitudinal Association of Baseline Dual Sensory Impairment With Incident Cognitive Decline.**

Legend: The gray diamonds are the estimated pooled Hazard Ratio (HR) for each random-effects meta-analysis; gray box sizes reflect the relative weight apportioned to studies in the meta-analysis.

Supplemental Figure S13**: Contour-Enhanced Funnel Plot for The Longitudinal Association of Baseline Dual Sensory Impairment With Incident Cognitive Decline, With Missing Studies Imputed Using The Trim-And-Fill Method.**

Legend: The shaded circles represent the original study estimates and the unshaded circle represents the missing estimate imputed via the trim-and-fill method. The dark gray, gray and light gray contour lines indicate conventional milestones in levels of statistical significance (P<0.1, 0.05, 0.01).

# SUPPLEMENTAL TABLES

Supplemental Table S1**: PRISMA Checklist.**

| **Section and Topic** | **Item #** | **Checklist Item** | **Page # Where Item is Reported** |
| --- | --- | --- | --- |
| **TITLE** | | |  |
| Title | 1 | Identify the report as a systematic review. | 1 |
| **ABSTRACT** | | |  |
| Abstract | 2 | See the PRISMA 2020 for abstract’s checklist. | 3 |
| **INTRODUCTION** | | |  |
| Rationale | 3 | Describe the rationale for the review in the context of existing knowledge. | 4 |
| Objectives | 4 | Provide an explicit statement of the objective(s) or question(s) the review addresses. | 4 |
| **METHODS** | | |  |
| Eligibility Criteria | 5 | Specify the inclusion and exclusion criteria for the review and how studies were grouped for the syntheses. | 5 |
| Information Sources | 6 | Specify all databases, registers, websites, organisations, reference lists and other sources searched or consulted to identify studies. Specify the date when each source was last searched or consulted. | 5 |
| Search Strategy | 7 | Present the full search strategies for all databases, registers and websites, including any filters and limits used. | 5 |
| Selection Process | 8 | Specify the methods used to decide whether a study met the inclusion criteria of the review, including how many reviewers screened each record and each report retrieved, whether they worked independently, and if applicable, details of automation tools used in the process. | 5 |
| Data Collection Process | 9 | Specify the methods used to collect data from reports, including how many reviewers collected data from each report, whether they worked independently, any processes for obtaining or confirming data from study investigators, and if applicable, details of automation tools used in the process. | 6 |
| Data Items | 10a | List and define all outcomes for which data were sought. Specify whether all results that were compatible with each outcome domain in each study were sought (e.g. for all measures, time points, analyses), and if not, the methods used to decide which results to collect. | 6 |
|  | 10b | List and define all other variables for which data were sought (e.g. participant and intervention characteristics, funding sources). Describe any assumptions made about any missing or unclear information. | 6 |
| Study Risk of Bias Assessment | 11 | Specify the methods used to assess risk of bias in the included studies, including details of the tool(s) used, how many reviewers assessed each study and whether they worked independently, and if applicable, details of automation tools used in the process. | 6 |
| Effect Measures | 12 | Specify for each outcome the effect measure(s) (e.g. risk ratio, mean difference) used in the synthesis or presentation of results. | 7 |
| Synthesis Methods | 13a | Describe the processes used to decide which studies were eligible for each synthesis (e.g. tabulating the study intervention characteristics and comparing against the planned groups for each synthesis (item #5)). | 7 |
|  | 13b | Describe any methods required to prepare the data for presentation or synthesis, such as handling of missing summary statistics, or data conversions. | 7 |
|  | 13c | Describe any methods used to tabulate or visually display results of individual studies and syntheses. | 7 |
|  | 13d | Describe any methods used to synthesize results and provide a rationale for the choice(s). If meta-analysis was performed, describe the model(s), method(s) to identify the presence and extent of statistical heterogeneity, and software package(s) used. | 7 |
|  | 13e | Describe any methods used to explore possible causes of heterogeneity among study results (e.g. subgroup analysis, meta-regression). | 7 |
|  | 13f | Describe any sensitivity analyses conducted to assess robustness of the synthesized results. | 7 |
| Reporting Bias Assessment | 14 | Describe any methods used to assess risk of bias due to missing results in a synthesis (arising from reporting biases). | 7 |
| Certainty Assessment | 15 | Describe any methods used to assess certainty (or confidence) in the body of evidence for an outcome. | 7 |
| **RESULTS** | | |  |
| Study Selection | 16a | Describe the results of the search and selection process, from the number of records identified in the search to the number of studies included in the review, ideally using a flow diagram. | 8 |
|  | 16b | Cite studies that might appear to meet the inclusion criteria, but which were excluded, and explain why they were excluded. | 8 |
| Study Characteristics | 17 | Cite each included study and present its characteristics. | 8 |
| Risk of Bias in Studies | 18 | Present assessments of risk of bias for each included study. | 8 |
| Results of Individual Studies | 19 | For all outcomes, present, for each study: (a) summary statistics for each group (where appropriate) and (b) an effect estimate and its precision (e.g. confidence/credible interval), ideally using structured tables or plots. | 8 |
| Results of Syntheses | 20a | For each synthesis, briefly summarise the characteristics and risk of bias among contributing studies. | 8 |
|  | 20b | Present results of all statistical syntheses conducted. If meta-analysis was done, present for each the summary estimate and its precision (e.g. confidence/credible interval) and measures of statistical heterogeneity. If comparing groups, describe the direction of the effect. | 8 |
|  | 20c | Present results of all investigations of possible causes of heterogeneity among study results. | 8 |
|  | 20d | Present results of all sensitivity analyses conducted to assess the robustness of the synthesized results. | 8 |
| Reporting Biases | 21 | Present assessments of risk of bias due to missing results (arising from reporting biases) for each synthesis assessed. | 8 |
| Certainty of Evidence | 22 | Present assessments of certainty (or confidence) in the body of evidence for each outcome assessed. | 11 |
| **DISCUSSION** | | |  |
| Discussion | 23a | Provide a general interpretation of the results in the context of other evidence. | 12 |
|  | 23b | Discuss any limitations of the evidence included in the review. | 14 |
|  | 23c | Discuss any limitations of the review processes used. | 14 |
|  | 23d | Discuss implications of the results for practice, policy, and future research. | 14 |
| **OTHER INFORMATION** | | |  |
| Registration and Protocol | 24a | Provide registration information for the review, including register name and registration number, or state that the review was not registered. | 5 |
|  | 24b | Indicate where the review protocol can be accessed, or state that a protocol was not prepared. | 5 |
|  | 24c | Describe and explain any amendments to information provided at registration or in the protocol. | 5 |
| Support | 25 | Describe sources of financial or non-financial support for the review, and the role of the funders or sponsors in the review. | 25 |
| Competing Interests | 26 | Declare any competing interests of review authors. | 25 |
| Availability of Data, Code and Other Materials | 27 | Report which of the following are publicly available and where they can be found: template data collection forms; data extracted from included studies; data used for all analyses; analytic code; any other materials used in the review. | 25 |

Supplemental Table S2**: Study Characteristics of Included Studies.**

| **First Author Year** | **Study Name** | **Country** | **Study Design** | **Definition of DSI** | **Measurement of Hearing Parameters** | **Definition of Hearing Impairment** | **Severity of Hearing Impairment** | **Measurement of Vision Parameters** | **Definition of Vision Impairment** | **Severity of Vision Impairment** | **Primary Measurement of Cognition** | **Definition of Cognitive Impairment, Cognitive Decline or Dementia** |
| --- | --- | --- | --- | --- | --- | --- | --- | --- | --- | --- | --- | --- |
| Armstrong et al. 2022 (1) | Health Aging and Body Composition Study | USA | Prospective Cohort | Visual and Hearing Impairment | Pure-Tone Audiometry | PTA > 25 dB at 0.5, 1, 2, 4 kHz for Better Hearing Ear | - | Visual Acuity: Bailey-Lovie Test  Contrast Sensitivity: Pelli-Robson Letter Chart | Visual Acuity ≤ 20/50 or Contrast Sensitivity ≤ 1.3 Units | - | - | - |
| Armstrong et al. 2022 (2) | Baltimore Longitudinal Study of Aging; Atherosclerosis Risk in Communities Study | USA | Retrospective Cohort | Visual and Hearing Impairment | Pure-Tone Audiometry | PTA > 25 dB at 0.5, 1, 2, 4 kHz for Better Hearing Ear | - | Visual Acuity: Early Treatment of Diabetic Retinopathy Study Chart R Contrast Sensitivity: Pelli-Robson Letter Charts Visual Field: Bilateral Visual Field Test Stereo Acuity: Randot Stereo Test | Visual Acuity < 20/40 or Log Contrast Sensitivity < 1.55 Units or Visual Field > 1 Standard Deviation of Population Mean Score (/96) or Stereo Acuity > 80 Arcseconds | - | - | - |
| Bikbov et al. 2022 | The Ural Very Old Study | Russia | Cross Sectional Study | Moderately Severe Vision Impairment or Blindness and Moderately Severe or Severe Hearing Impairment | Hearing Handicap Inventory for The Elderly Screening Version (HHIE-S) | Mild HI: HHIE-S 11-17 Moderate HI: HHIE-S 18-24 Moderately Severe HI: HHIE-S 25-31 Severe HI: HHIE-S 32-38 Profound HI: HHIE-S 39-44 | Mild HI: 55 (7.5%) Moderate HI: 143 (19.6%) Moderately Severe HI: 66 (9.0%) Severe HI: 58 (7.9%) Profound HI: 118 (16.1%) | Best Correct Visual Acuity (BCVA) Score | Moderate-Severe VI: BCVA Score < 6/18 but ≥ 3/60 in Better Eye or Binocularly; Blindness: BCVA Score < 3/60 in Better Eye or Binocularly | Moderate-Severe VI: 342 (46.8%) Blindness: 37 (5.1%) | Mini-Mental State Examination (MMSE) | Cognitive Impairment: MMSE < 24 |
| Byeon et al. 2021 | Korean Longitudinal Study on Cognitive Aging and Dementia | South Korea | Prospective Cohort Study | Visual and Hearing Impairment | Self-Reported Assessment 0: “normal”. 1: “reduced but able to communicate without using a hearing aid”.  2: “reduced so that communication is possible only using hearing aids”. 3: “difficulty communicating even when hearing aid is used” 4: “i can’t hear at all”. | Self-Reported Assessment 1-4/4 | - | Self-Reported Assessment 0: “normal”. 1: “reduced but able to view newspaper or television without wearing glasses or lenses”.  2: “reduced, so that i can view newspaper or television only with glasses or lenses”. 3: “reduced, as a result, i can’t view newspaper or television even if i wear glasses of lenses”. 4: “i can’t see at all”. | Self-Reported Assessment 1-4/4 | - | Korean Version of Consortium to Establish a Registry for Alzheimer's Disease Assessment Packet (CERAD-K) | Mild Cognitive Impairment: Consensus Criteria from the International Working Group on Mild Cognitive Impairment  Dementia: DSM-IV  Alzheimer’s Disease: National Institute of Neurologic and Communicative Disorders and Stroke and the Alzheimer’s Disease and Related Disorders Association  Vascular Dementia**:** National Institute of Neurologic Disorders and Stroke–Association Internationale pour la Recherche et l’Enseignement en Neu- rosciences Neurocognitive Tests |
| Cai et al. 2023 | Baltimore Longitudinal Study of Ageing | USA | Prospective Cohort Study | Visual and Hearing Impairment | Pure-Tone Audiometry | PTA > 25 dB at 0.5, 1, 2, 4 kHz for Better Hearing Ear | - | Visual Acuity: Early Treatment of Diabetic Retinopathy Study Eye Chart  Contrast Sensitivity: Pelli-Robson Chart Binocularly with Corrective Lenses  Visual Field: Humphrey 81-Point Single Intensity (24 dB) Full Field Test Monocularly, Binocular Vision Field Calculated from Composite Monocular Values  Stereo Acuity: Randot Stereo Vision Test | Visual Acuity < 20/40 in Better Eye on ETDRS Eye Chart or Contrast Sensitivity Log of Contrast Units < 1.55 on Pelli-Robson Chart or Visual Fields (Number of Missed Points >1 SD of Population Mean Out of 96 Points or Stereo Acuity Minimum Depth Differential > 80s or Arc | - | Card Rotation Test and Immediate Free Recall of the California Verbal Learning Test | Early Cognitive Impairment: ≥ 1 Standard Deviation Below Age, Sex, Race, Education Specific Means in Cohort Data |
| Davidson et al. 2019 | InterRAI Data from Ontario | Canada | Retrospective Cohort Study | Deaf-Blind Severity Index Score ≥ 3 | Self-Reported Assessment  0: Adequate Hearing  1: Mild HI (difficulties when not in a quiet setting)  2: Moderate HI (speaker has to adjust quality/tone of speech)  3: Severe HI (absence of useful hearing) | Self-Reported Assessment 1-3/3 | Mild HI: 38498 Moderate HI: 29443 Severe HI: 2247 | Self-Reported Assessment  0: Adequate Vision  1: Mild VI (problems reading regular sized print like newspapers)  2: Moderate VI (inability to see large print like newspaper headlines)  3: High VI (problem identifying objects but the client was able to follow objects)  4: Severe VI (no vision) | Self-Reported Assessment 1-4/4 | Mild VI: 46785 Moderate VI: 15078 High VI: 7539 Severe VI: 2786 | Cognitive Performance Scale (CPS) | Cognitive Impairment: CPS Score ≥ 2/6 |
| Dawes et al. 2014 | UK Biobank Study | United Kingdom | Retrospective Cohort Study | Visual and Hearing Impairment | Speech Reception Threshold using Digit Triplet Test | Lower Speech Reception Threshold Value ≤ -5.5 dB in Better Ear | - | Lower Logarithm of Minimum Angle of Resolution (LogMAR) in Better Eye | < 0.8 LogMAR Units  Mild VI: 0.3 ≤ LogMAR < 0.8 Low Vision: LogMAR < 0.3 |  | - | - |
| Dintica et al. 2023 | Swedish National Study on Aging and Care Kungsholmen | Sweden | Prospective Cohort Study | Visual and Hearing Impairment | Self-Reported Assessment (using the question “do you have trouble hearing”, with plausible answers as a) yes, but uses hearing aid, b) yes, but uses no aid and c) no) or Objectively Diagnosed by ICD Codes | Previous Diagnosis of Hearing Loss, or Self-Reported "Trouble Hearing" | - | Visual Acuity at Normal Reading Distance: Jaeger Eye Chart Test | Reading Acuity < 20/20 (≥ J2 on Jaegar Eye Chart) | - | Mini-Mental State Examination (MMSE) | Dementia: Clinician-Led Clinical Diagnosis based on DSM-IV-TR |
| Fisher et al. 2014 | AGES-Reykjavik Study | Iceland | Prospective Cohort Study | Visual and Hearing Impairment | Pure-Tone Audiometry | PTA Average ≥ 35dB at 0.5, 1, 2, 4 kHz for Better Hearing Ear | - | Table-Top Nidek ARK 760A Autorefractor with Built-In Acuity Charts | Visual Acuity ≤ 20/50 in Better Eye | - | - | Cognitive Impairment: Professional Consensus After Reviewing Results of Cognitive Examinations |
| Garcia-Morales et al. 2023 | National Health and Aging Trends Study | USA | Retrospective Cohort Study | Visual and Hearing Impairment | Pure-Tone Audiometry | PTA > 25 dB at 0.5, 1, 2, 4 kHz for Better Hearing Ear  Mild HI: 25dB < PTA ≤ 40dB Moderate HI: 40dB < PTA ≤ 60dB Severe HI: PTA > 60dB | Mild HI:  Moderate HI:  Severe HI: | Computation of Logarithm of Minimum Angle of Resolution for Distance (LogMARD), Logarithm of Minimum Angle of Resolution for Near Acuity (LogMARN), and Logarithm of Contrast Sensitivity (LogCS) | LogMARD > 0.30 Units or LogMARN > 0.30 Units or LogCS < 1.55 Units | - | - | - |
| Ge et al. 2021 | Health and Retirement Study and Supplement: The Aging, Demographics, and Memory Study (ADAMS) | USA | Retrospective Cohort Study | Visual and Hearing Impairment | Pure-Tone Audiometry | PTA > 25 dB at 0.5, 1, 2, 4 kHz for Better Hearing Ear | - | Snellen Chart | Corrected Binocular Vision < 20/40 | - | Telephone Interview for Cognitive Status (TICS) | - |
| Guthrie et al. 2016 | Data Collected from InterRAI Data from Ontario, State of Michigan Medicaid Home Care System, BelRAI Database, Finnish RAI Database | Canada, USA, Finland, Belgium | Cross Sectional Study | Deafblind Severity Index Score ≥ 3 | Self-Reported Assessment  0: Adequate Hearing  1: Mild HI (difficulties when not in a quiet setting)  2: Moderate HI (speaker has to adjust quality/tone of speech)  3: Severe HI (absence of useful hearing) | Self-Reported Assessment 1-3/3 | - | Self-Reported Assessment  0: Adequate Vision  1: Mild VI (problems reading regular sized print like newspapers)  2: Moderate VI (inability to see large print like newspaper headlines)  3: High VI (problem identifying objects but the client was able to follow objects)  4: Severe VI (no vision) | Self-Reported Assessment 1-4/4 | - | Cognitive Performance Scale (CPS) | Cognitive Impairment: CPS Score ≥ 1/6 |
| Guthrie et al. 2022 | InterRAI Assessment | USA | Retrospective Cohort Study | Deafblind Severity Index Score ≥ 3 | Self-Reported Assessment  0: Adequate Hearing  1: Mild HI (difficulties when not in a quiet setting)  2: Moderate HI (speaker has to adjust quality/tone of speech)  3: Severe HI (absence of useful hearing) | Self-Reported Assessment 1-3/3 | - | Self-Reported Assessment  0: Adequate Vision  1: Mild VI (problems reading regular sized print like newspapers)  2: Moderate VI (inability to see large print like newspaper headlines)  3: High VI (problem identifying objects but the client was able to follow objects)  4: Severe VI (no vision) | Self-Reported Assessment 1-4/4 | - | Cognitive Performance Scale (CPS) | Cognitive Impairment: CPS Score ≥ 2/6 |
| Harada et al. 2008 | - | Japan | Cross Sectional Study | Visual and Hearing Impairment | Pure-Tone Audiometry | PTA > 30 dB at 1 kHz Bilaterally | - | Corrected Visual Acuity using Landolt Broken Ring Chart at 5 Meters using Automatic Visual Analyzer | Corrected Visual Acuity < 0.5 in Better Eye | - | - | - |
| Harithasan et al. 2020 | Long Term Research Grant Study | Malaysia | Cross Sectional Study | Visual and Hearing Impairment | Pure-Tone Audiometry | PTA > 40 dB at 0.5, 1, 2, 4 kHz for Better Hearing Ear  Mild HI: 25dB < PTA ≤ 40dB Moderate HI: 40dB < PTA ≤ 70dB Severe HI: PTA > 70dB | Normal: 20 Mild HI: 144  Severe HI: 65 | Lighthouse International Chart (Literate Participants) or Tumbling E (Participants Unable to Read or Write) | VI: < 0.3 LogMAR Units (Snellen 20/40) in The Better Eye | Normal: 164 Mild VI: 51 Severe VI: 14 | Mini-Mental State Examination (MMSE) Malay Version | Cognitive Impairment: Modified MMSE (Malay Version) Score < 22 |
| Hong et al. 2016 | Blue Mountains Eye Study | Australia | Retrospective Cohort Study | Visual and Hearing Impairment | Pure-Tone Audiometry | PTA > 40 dB at 0.5, 1, 2, 4 kHz for Worse Hearing Ear (Primary Analysis)/ Better Hearing Ear (Supplementary Analysis) | - | Retro-Illuminated Logarithm of Minimum Angle of Resolution (LogMAR) | Best-Corrected Visual Acuity after Subjective Refraction < 6/12 (39 Letters or Less Read) in Worse Eye (Primary Analysis)/ Better Eye (Supplementary Analysis) | - | Mini-Mental State Examination (MMSE) Blind | Cognitive Decline: Decline of ≥ 3 MMSE Blind Scores between 5-Year and 10- or 15-Year Follow-Up Visits |
| Hu et al. 2022 | UK Biobank Study | United Kingdom | Retrospective Cohort Study | Visual and Hearing Impairment | Speech Reception Threshold Using Digit Triplet Test | Lower Speech Reception Threshold Value ≤ -5.5dB in Better Ear | - | Lower Logarithm of Minimum Angle of Resolution (LogMAR) in Better Eye | < 0.3 LogMAR Units (Snellen 20/40) in Better Eye | - | International Classification of Diseases (ICD) 9/10 | Dementia: International Classification of Diseases (ICD) 9/10 All-Cause Dementia |
| Hwang et al. 2020 | Gingko Evaluation of Memory Study | USA | Retrospective Cohort Study | Visual and Hearing Impairment | Self-Reported Assessment  1: “can you hear well enough (with or without) a hearing aid to listen to the radio”.  2: “can you hear well enough (with or without) a hearing aid to use the telephone”.  3: “can you hear well enough (with or without) a hearing aid to carry on a conversation in a crowded room”. | Negative Response ≥ 1 of the Assessment Questions | Low DSI Score (1-2): 62 Moderate DSI Score (3-4): 30 Severe DSI Score (5-6): 12 | Self-Reported Assessment  1: “can you see well enough (with or without) glasses to drive”.  2: “can you see well enough (with or without) glasses to watch television”.  3: “can you see well enough (with or without) glasses to read the newspaper”.  4: “can you see well enough (with or without) glasses to recognize someone across the room”. | Negative Response ≥ 1 of the Assessment Questions | Low DSI Score (1-2): 62 Moderate DSI Score (3-4): 30 Severe DSI Score (5-6): 12 | Modified Mini-Mental State Examination (3MSE), Clinical Dementia Rating (CDR) Scale and Cognitive Subscale of the Alzheimer’s Disease Assessment Scale (ADAS-Cog) | Dementia: Decline on Broad Range of Test Scores (3MSE, CDR, ADAS-Cog) and Categorised According to DSM-IV Criteria |
| Hwang et al. 2022 | Cardiovascular Health Study | USA | Prospective Cohort Study | Visual and Hearing Impairment at Same Visit | Self-Reported Assessment  1: “can you hear well enough (with or without) a hearing aid to listen to the radio”.  2: “can you hear well enough (with or without) a hearing aid to use the telephone”.  3: “can you hear well enough (with or without) a hearing aid to carry on a conversation in a crowded room”. | Negative Response ≥ 1 of the Assessment Questions | - | Self-Reported Assessment  1: “can you see well enough (with or without) glasses to drive”.  2: “can you see well enough (with or without) glasses to watch television”.  3: “can you see well enough (with or without) glasses to read the newspaper”.  4: “can you see well enough (with or without) glasses to recognize someone across the room”. | Negative Response ≥ 1 of the Assessment Questions | - | Modified Mini-Mental State Examination (3MSE) | Mild Cognitive Impairment: Failing Cognitive Test Scores (MMSE) but Do Not Meet DSM-IV Criteria  Dementia: Consensus of a Panel of Neurologists and Psychiatrists Using Results from Neuropsychiatric Tests Based on DSM-IV Criteria |
| Jee et al. 2005 | Age Care Clients Study | Australia | Cross-Sectional Study | Visual and Hearing Impairment | Pure-Tone Audiometry | PTA > 25 dB at 0.5, 1, 2, 4 kHz for Better Hearing Ear  Mild HI: 25dB < PTA ≤ 40dB Moderate HI: 40dB < PTA ≤ 60dB Severe HI: PTA > 60dB | - | LogMAR Chart R With Standard Illumination at 2.4 Metres | Visual Acuity <20/40 in Better Eye  Mild VI: 20/80 ≤ VA <20/40 or 24-38 Letters Read Correctly Moderate VI: 20/200 ≤ VA < 20/80 or 4-23 Letters Read Correctly Severe VI: VA < 20/200 or < 4 Letters Read Correctly | - | - | - |
| Joo et al. 2022 | Korean Longitudinal Study of Aging | South Korea | Retrospective Cohort Study | Visual and Hearing Impairment | Self-Reported Assessment, Graded as “Excellent”, “Very Good”, “Good”, “Fair” or “Poor”. | Self-Reported Assessment Reported as "Fair" or "Poor" | - | Self-Reported Assessment, Graded as “Excellent”, “Very Good”, “Good”, “Fair” or “Poor”. | Self-Reported Assessment Reported as "Fair" or "Poor". | - | Mini-Mental State Examination (MMSE) Korean Version | Cognitive Impairment: Korean MMSE Score < 24 |
| Jung et al. 2022 | Korean National Health Insurance Service Database -National Health Screening Program 2009 | South Korea | Prospective Cohort Study | Visual and Hearing Impairment | Pure-Tone Audiometry | PTA ≥ 40dB in ≥ 1 Ear or Hearing Disability in The National Disability Registry | - | Visual Acuity Measurement | Visual Acuity < 20/40 in Both Eyes or Visual Disability in The National Disability Registry | - | - | - |
| Khil et al. 2015 | Dortmund Health Study | Germany | Cross Sectional Study | Visual and Hearing Impairment | Pure-Tone Audiometry | Average PTA Over 3 Frequencies (1, 2, 4 kHz) >30 dB In Worse Ear | - | Standard Snellen Chart at 4 Metres | Monocular Visual Acuity <4/7.5 In Worse Eye | - | - | - |
| Kim et al. 2015 | Korea National Health and Nutrition Examination Survey | South Korea | Cross Sectional Study | Visual and Hearing Impairment | Pure-Tone Audiometry | PTA > 40 dB at 0.5, 1, 2, 4 kHz for Better Hearing Ear | - | Visual Acuity Using LogMAR Test for Corrected Vision of Both Eyes | Maximum Corrected Vision < 0.33 in One or Both Eyes (<6/18) | - | - | - |
| Kulmala et al. 2009 | Finnish Twin Study on Aging | Finland | Prospective Cohort Study | Visual and Hearing Impairment | Pure-Tone Audiometry | PTA ≥ 21 dB at 0.5, 1, 2, 4 kHz for Better Hearing Ear | - | Best Corrected Visual Acuity Measured at 5 Metres With and Without Participants' Spectacles Using The Illuminated Landolt Ring Chart | Best Corrected Visual Acuity < 1.0 | - | - | - |
| Liang et al. 2023 | - | China | Cross Sectional Study | Visual and Hearing Impairment | Self-Reported Assessment - “can you hear someone in the room speaking in a normal voice” (yes or no). | Self-Reported Assessment Reported as “Yes” | - | Self-Reported Assessment - “do you suffer from poor vision when you do daily activities such as reading books or watching television” (yes or no). | Self-Reported Assessment Reported as “Yes” | - | Mini-Mental State Examination (MMSE) | Cognitive Impairment: MMSE ≤ 24 (education level of junior high and above) MMSE ≤ 20 (education level of primary school) MMSE ≤ 17 (no education) |
| Lin et al. 2004 | Study of Osteoporotic Fractures | USA | Prospective Cohort Study | Visual and Hearing Impairment | Hand-Held Audiometer Hearing Test in A Quiet Room Without the Use of Hearing Aids | Inability to Hear a Tone ≥ 40dB at 2 kHz in Better Ear | - | Binocular Visual Acuity Test Using Standard Protocol and Bailey Love Targets Using Standardized Illumination of 50-Foot to 70-Foot Lamberts with Visual Correction | Corrected Binocular Vision < 20/40 | - | Modified Mini-Mental State Examination (3MS) | Cognitive Decline: Change in 3MS from Baseline to Follow-Up that Exceeds the Observed Average Change in Scores by > 1 SD |
| Loprinzi et al. 2013 | National Health and Nutrition Examination Survey | USA | Cross Sectional Study | Visual and Hearing Impairment | Pure-Tone Audiometry | HI: PTA ≥ 25 dB at Low Frequency PTA (0.5, 1, 2 kHz) or High Frequency PTA (3, 4, 6, 8 kHz) for Better Hearing Ear | - | Visual Acuity. If Presenting Visual Acuity < 20/30, Corrected Lenses were Removed (if worn) and Objective Refraction was Measured Using Autorefractor | Visual Acuity < 20/40 After Autorefraction or Unable to See Light with Both Eyes Open | - | - | - |
| Lundin et al. 2020 | - | Sweden | Cross Sectional Study | Moderate Visual Impairment and Severe Hearing Impairment | Pure-Tone Audiometry | PTA ≥ 70 dB at 0.5, 1, 2, 4 kHz for Better Hearing Ear | - | - | Decimal Visual Acuity ≤ 0.3 for Distance in Better Eye with Best Correction | - | - | - |
| Luo et al. 2018 | Second National Sample Survey on Disability | China | Cross Sectional Study | Visual and Hearing Impairment | Pure Tone Audiometry | PTA > 40 dB at 0.5, 1, 2, 4 kHz for Better Hearing Ear | - | Best-Correct Visual Acuity (BCVA) | Low Vision: BCVA 0.05-0.29; Blindness: BCVA < 0.05; Visual Field < 10 Degrees in Better Seeing Eye | - | International Classification of Diseases (ICD) 10 | Dementia: Psychiatrists-Diagnosed Dementia Based on International Classification of Diseases (ICD) 10 |
| Lyu et al. 2018 | Korean Longitudinal Study of Ageing | South Korea | Cross Sectional Study | Visual and Hearing Impairment | Self-Reported Assessment, Graded as “Very Good”, “Good”, “Fair”, “Poor” or “Very Poor” | Self-Reported Assessment Reported as "Poor" or “Very Poor” | - | Self-Reported Assessment Graded as “Very Good”, “Good”, “Fair”, “Poor” or “Very Poor” | Self-Reported Assessment Reported as "Poor" or “Very Poor” | - | Mini-Mental State Examination Korean Version (K-MMSE) | Cognitive Impairment: K-MMSE Score Difference ≥ 2 SD from Mean of K-MMSE Normative Data |
| Mah et al. 2020 | Grand Challenge Project | Malaysia | Cross Sectional Study | Visual and Hearing Impairment | Pure-Tone Audiometry | PTA >25 dB at 0.5, 1, 2, 4 kHz for Better Hearing Ear | - | Early Treatment Diabetic Retinopathy Study Chart, Logarithm of Minimum Angle of Resolution (LogMAR) in Better Eye | Habitual Distance Visual Acuity > 0.3 LogMAR Units in Better Eye | - | Montreal Cognitive Assessment Malay Version (MoCA-BM) | Cognitive Impairment: MoCA-BM Score < 17/18 |
| Maharani et al. 2020 | Health and Retirement Study | USA | Retrospective Cohort Study | Visual and Hearing Impairment | Self-Reported Assessment, Graded as “Excellent”, “Very Good”, “Good”, “Fair” or “Poor” | Self-Reported Assessment Reported as "Fair" or "Poor" | - | Self-Reported Assessment, Graded as “Excellent”, “Very Good”, “Good”, “Fair” or “Poor” | Self-Reported Assessment Reported as "Fair" or "Poor" | - | Adapted Telephone Interview for Cognitive Status (TICS) | Probable Dementia: TICS Score 0-6/27.  Possible Cognitive Impairment, No Dementia: TICS 7-11/27. |
| Maruta et al. 2020 | Long-Term Care Insurance System | Japan | Retrospective Cohort Study | Visual and Hearing Impairment | Self-Reported Assessment Evaluated at 5 Levels  1: ‘normal hearing (there is no hindrance in daily life)’.  2: ‘hardly catch normal conversation’.  3: ‘hardly catch loud conversation’.  4: ‘hardly hear’.  5: ‘undecidable due to difficulty in communication’. | Self-Reported Assessment Reported as The Following Answers: ‘hardly catch normal conversation,’ ‘hardly catch loud conversation,’ ‘hardly hear,’ | - | Self-Reported Assessment Evaluated at 5 Levels  1: ‘normal vision (there is no hindrance in daily life)’  2: ‘able to see vision testing chart at a distance of about one meter’  3: ‘able to see vision testing chart at a distance of in front’  4: ‘very poor eyesight’  5: ‘undecidable due to difficulty in communication’. | Self-Reported Assessment Reported as The Following Answers: ‘able to see vision testing chart at a distance of about one meter,’ ‘able to see vision testing chart at a distance of in front,’ ‘very poor eyesight’ | - | Dementia Scale Developed by The Japanese Ministry of Health, Labour and Welfare | Dementia: Dementia Scale Developed by The Japanese Ministry of Health, Labour and Welfare to be Exceeding Level 1 in Which Patient "Has Some Dementia, but can Live Independently at Home and in Society" |
| Mitoku et al. 2016 | Gujo City Long-Term Care Insurance Database | Japan | Retrospective Cohort Study | Visual and Hearing Impairment | Self-Reported Assessment Evaluated at 5 Levels, Confirmed by ICD-10  1: “normal hearing”.  2: “barely hear normal conversation”.  3: “barely hear loud conversation”.  4: “barely hear”.  5: “indeterminable due to communication difficulty”. (“barely hear normal conversation” and “barely hear loud conversation” were merged to form one value “able to hear loud conversation” during analysis) | Inability to Hear Loud Conversations, Diagnoses Confirmed by ICD-10 Diagnosed HI | - | Self-Reported Assessment, Evaluated at 5 Levels, Confirmed by ICD-10  1: “normal sight”.  2: “able to see a visual acuity chart at a distance of one meter”.  3: “able to see a visual acuity chart in front”.  4: “very little sight”.  5: “indeterminable due to communication difficulty”.  (“able to see a visual acuity chart at a distance of one meter” and “able to see a visual acuity chart in front” were merged to form one value “able to see an object near the front of the eyes” during analysis). | Inability To See an Object Near the Front of Eyes, Diagnoses Confirmed by ICD-10 Diagnosed VI | - | International Classification of Diseases (ICD) 10 | Mild-Severe Cognitive Impairment: Assessed by Trained and Certified Investigators using The Functional Assessment for Cognitively Impaired Elders, Defined by Requiring Assistance (Moderate Cognitive Impairment) due to Symptoms Related to Cognitive Impairment, or Requiring Care (Moderate Cognitive Impairment) due to Symptoms Related to Cognitive Impairment, Confirmed by International Classification of Diseases ICD-10 |
| Oh et al. 2023 | Korean National Health Insurance Service Database -National Health Screening Program 2011 | South Korea | Retrospective Cohort Study | Hearing and Visual Impairment | Pure-Tone Audiometry | Inability to Hear Sounds ≥ 40dB at 1 kHz in Both Ears | - | Snellen Chart | Visual Acuity < 0.3 In Both Eyes, Even After Vision Correction with Glasses or Contact Lens | - | ICD-10 | Dementia: ICD-10 Diagnosis of Dementia |
| Pabst et al. 2021 | LEILA75+ Study and AgeCode Study | Germany | Prospective Cohort Study | Hearing and Visual Impairment | Self-Reported Assessment - “do you have difficulty hearing” ((1) no impairment, (2) slight impairment, (3) moderate impairment, and (4) severe/profound impairment) | Self-Reported Assessment to Have "Difficulty Hearing" | - | Self-Reported Assessment - “do you have difficulty seeing” ((1) no impairment, (2) slight impairment, (3) moderate impairment, and (4) severe/profound impairment) | Self-Reported Assessment to Have "Difficulty Seeing" | - | Mini-Mental State Examination (MMSE) | Dementia: Diagnosed by Various DSM Criteria Upon Assessment Based on The Structured Interview for The Diagnosis of The Alzheimer Type, Multi-Infarct Dementia, And Dementia of Other Etiology (SIDAM) DSM-III-R Criteria in The LEILA75+ Study, DSM-IV Criteria in The AgeCode Study |
| Parada et al. 2021 | Rancho Bernardo Study of Healthy Aging | USA | Retrospective Cohort Study | Visual and Hearing Impairment | Pure-Tone Audiometry | PTA > 25 dB at 0.5, 1, 2, 4 kHz for Better Hearing Ear | - | Bailey-Lovie Chart | Vision < 20/40 in The Better Eye (LogMAR > 0.30) | - | Mini-Mental State Examination (MMSE), Verbal Fluency Test, Trials B | Mild Cognitive Impairment: > 2.0 Standard Deviations Below Sex, Age and Education-Adjusted Mean for MMSE Based on Normative Data from The National Alzheimer’s Coordinating Centre Uniform Data Set |
| Schneck et al. 2012 | Smith-Kettlewell Institute Study | USA | Cross Sectional Study | Visual and Hearing Impairment | Pure-Tone Audiometry | PTA > 40 dB at 0.5, 2, 4 kHz for Either Ear | - | Test Battery: Bailey-Lovie Chart, Berkeley Glare Test, SKILL Card Dark Chart, Pelli-Robson Chart, Frisby Stereo Test, Farnsworth-Munsell D-15 Test under MacBeth Lamp Illumination | Binocular Visual Acuity < 0.54 LogMAR, Snellen Chart Equivalent 6/21 or 20/70 | - | - | - |
| Schneider et al. 2012 | Blue Mountains Eye Study | Australia | Prospective Cohort Study | Visual and Hearing Impairment | Pure-Tone Audiometry | HI: PTA > 25 dB at 0.5, 1, 2, 4 kHz for Better Hearing Ear  Moderate-Severe HI: > 40dB | - | Retro-Illuminated Logarithm of Minimum Angle of Resolution (LogMAR) | Visual Acuity of Better Eye < 20/40 | - | - | - |
| Tomida et al. 2022 | National Centre for Geriatrics and Gerontology-Study on Geriatric Syndrome | Japan | Cross Sectional Study | Visual and Hearing Impairment | Hearing Handicap Inventory for the Elderly-Screening (HHIE-S) | HHIE-S Score > 8 Points | - | Visual Function Index 14 (VF-14) Questionnaire | VF-14 Score < 75 | - | Mini-Mental State Examination (MMSE), National Centre for Geriatrics and Gerontology-Functional Assessment Tool (NCGG-FAT) | Cognitive Impairment: ≤ 1.5 Standard Deviations Below Age and Education-Specific Mean Derived from Healthy Older Adults |
| Wong et al. 2023 | US California Medicare Beneficiaries Database | USA | Cross Sectional Study | Visual and Hearing Impairment (ICD-9 Code V49.85 or ICD-10 Code Z73.82) | ICD-9/10 | ICD-9 Codes 389.X or ICD-10 Codes H90.X or H91.X | - | ICD-9/10 | ICD-9 Codes 369.X or ICD-10 H54.X | - | - | - |
| Yamada et al. 2016 | Services and Health for Elderly in Long Term Care | Czech Republic, England, Finland, France, Germany, Israel, Italy, and the Netherlands | Prospective Cohort Study | Visual and Hearing Impairment | Self-Reported Assessment  0: Adequate (suggesting no difficulty in normal conversation with hearing appliance normally used)  1: Minimum Impairment (suggesting difficulty in some environments)  2: Moderate Impairment (suggesting problem with hearing normal conversation, requiring quiet setting to hear well)  3: Severe Impairment (suggesting difficulty in all situations)  4: Cannot Hear Anything | Self-Reported Assessment Reporting Moderate Impairment, Severe Impairment or Cannot Hear Anything | - | Self-Reported Assessment  0: Adequate (being able to see fine details, including regular print in newspapers with glasses or with other visual appliance normally used)  1: Minimum Impairment (able to see large print but not regular print in newspapers)  2: Moderate Impairment (not able to see newspaper headlines but can identify objects)  3: Severe Impairment (object identification is in questions but eyes appear to follow objects)  4: No Vision Presents | Self-Reported Assessment Reporting Moderate Impairment, Severe Impairment or No Vision Presents | - | Cognitive Performance Scale (CPS) | Cognitive Impairment: CPS Score > 1/6 |

**Supplementary Table S3: Patient Characteristics of Included Studies.**

| **First Author, Year** | **Total Sample Size** | **Number of Patients with DSI** | **Mean Age (SD)** | **Number of Males** | **Number of Females** | **Ethnicity/Nationality** | **Community or Institutionalised Patients** | **Baseline Cognitive Status** | **Baseline Mean Primary Cognitive Test Scores (SD)** |
| --- | --- | --- | --- | --- | --- | --- | --- | --- | --- |
| Armstrong et al. 2022 (1) | 2102 | 491 | 74.0 (2.8) | 1012 | 1090 | Americans - White 1336, Black 766 | Community | - | - |
| Armstrong et al. 2022 (2) | 1383 | 500 | - | 543 | 840 | Americans - White 803, Black 543, Others 37 | Community | - | - |
| Bikbov et al. 2022 | 731 | 150 | 88.1 (2.7) | - | - | Russians - Russians 251, Tartaes 334, Bashkirs 83, Chuvash 25, Mari 5, Others 33 | Community | Both Baseline Cognitive Impairment and Baseline Cognitively Normal | 22.4 (6.4) |
| Byeon et al. 2021 | 6520 | 2631 | 72.6 (7.4) | 2805 | 3706 | South Koreans | Community | Both Baseline Cognitive Impairment and Baseline Cognitively Normal | - |
| Cai et al. 2023 | 414 | 83 | 74 (8.8) | - | - | Americans - White 226 | Community | Baseline Cognitively Normal | - |
| Davidson et al. 2019 | 352640 | 72188 | 81.4 (7.9) | 129711 | 222929 | Canadians | Institutionalised | Both Baseline Cognitive Impairment and Baseline Cognitively Normal | - |
| Dawes et al. 2014 | 116082 | 3617 | - | 53207 | 63475 | English - White 94.1%, Mixed 0.6%, Asian/Asian British 2.0%, Black/Black British 1.6%, Chinese 0.3%, Others 0.9%, Prefer Not to Answer 0.3%, Missing Data 0.2% | Community | - | - |
| Dintica et al. 2023 | 2579 | 310 | - | 967 | 1612 | Swedish | Community | Baseline Cognitively Normal | - |
| Fisher et al. 2014 | 4926 | 343 | 76.4 (5.5) | 236 | 2121 | Icelandic | Community | - | - |
| Garcia Morales et al. 2023 | 2579 | 787 | - | 1212 | 1367 | Americans - White 2138, Black 189, Hispanic 166, Others 86 | Community | - | - |
| Ge et al. 2021 | 295 | 52 | 81.5 (5.6) | 141 | 154 | Americans - White 236, Black 51, Others 8 | Community | Baseline Cognitively Normal | - |
| Guthrie et al. 2016 | 811647 | 137035 | - | 284867 | 526780 | Canadians, Americans, Belgians, Finnish | Institutionalised | Both Baseline Cognitive Impairment and Baseline Cognitively Normal | - |
| Guthrie et al. 2022 | 106920 | 22148 | - | 35885 | 71035 | Canadians | Institutionalised | Both Baseline Cognitive Impairment and Baseline Cognitively Normal | - |
| Harada et al. 2008 | 843 | 82 | - | 351 | 492 | Japanese | Community | - | - |
| Harithasan et al. 2020 | 229 | 19 | - | 107 | 122 | Malaysians - Malays 59, Chinese 122, Indians 48 | Community | Baseline Cognitively Normal | - |
| Hong et al. 2016 | 1883 | 93 | 69.3 (8.4) | 1076 | 807 | Australians | Community | Baseline Cognitively Normal | 19.3 (2.8) |
| Hu et al. 2022 | 113511 | 730 | 56.8 (8.1) | 51680 | 61831 | English - White 102090, Others 11421 | Community | Baseline Cognitively Normal | - |
| Hwang et al. 2020 | 2051 | 104 | 78.4 (3.1) | 1146 | 905 | Americans - White 96.1%, Black 3.9% | Community | Baseline Cognitively Normal | 91.9 (4.7) |
| Hwang et al. 2022 | 2927 | 120 | 74.7 (4.8) | 1223 | 1704 | Americans - White 80%, Black 20% | Community | Baseline Cognitively Normal | 91.9 (4.7) |
| Jee et al. 2005 | 49 | 11 | 82.4 (6.5) | 19 | 30 | Australians | Institutionalised |  |  |
| Joo et al. 2022 | 4844 | 54 | - | - | - | South Koreans | Community | Both Baseline Cognitive Impairment and Baseline Cognitively Normal | - |
| Jung et al. 2022 | 771128 | 8720 | 56.7 (11.5) | 484131 | 286997 | South Koreans | Community | - | - |
| Khil et al. 2015 | 1208 | 46 | 51.9 (13.8) | 564 | 644 | Germans | Community |  |  |
| Kim et al. 2015 | 3636 | 224 | 72.3 (0.5) | 1563 | 2073 | South Koreans | Community | - | - |
| Kulmala et al. 2009 | 428 | 40 | 68.5 (3.5) | 0 | 428 | Finnish | Community | - | - |
| Liang et al. 2023 | 3853 | 272 | 71.4 (7.3) | 1933 | 1920 | Chinese | Institutionalised | Both Baseline Cognitive Impairment and Baseline Cognitively Normal | - |
| Lin et al. 2004 | 6112 | - | 76.1 | 0 | 6112 | Americans | Community | Both Baseline Cognitive Impairment and Baseline Cognitively Normal | 24.6 |
| Loprinzi et al. 2013 | 1445 | 29 | 47.1 (95%CI: 45.9-48.2) | 715 | 730 | Americans - White 1094, Other 350 | Community | - | - |
| Lundin et al. 2020 | Study: 1257  Population Estimates: 127638 | 101 | Population Estimates: 88.0 (7.3) | - | - | Swedish | Community | - | - |
| Luo et al. 2018 | 250752 | 5277 | 72.9 | - | - | Chinese | Community | Both Baseline Cognitive Impairment and Baseline Cognitively Normal | - |
| Lyu et al. 2018 | 3851 | 224 | - | 1664 | 2167 | South Koreans | Community | Both Baseline Cognitive Impairment and Baseline Cognitively Normal | - |
| Mah et al. 2020 | 210 | 22 | 72.5 (5.5) | 90 | 120 | Malaysians - Malays 9.1%, Chinese 77.3%, Indian 13.6% | Community | Baseline Cognitive Impairment | 15.3 (7.5) |
| Maharani et al. 2020 | 19618 | 984 | 57.7 (6.2) | 8747 | 10871 | Americans | Community | Both Baseline Cognitive Impairment and Baseline Cognitively Normal | 10.2 |
| Maruta et al. 2020 | 2190 | 295 | 78.9 (6.1) | 452 | 1738 | Japanese | Community | Baseline Cognitively Normal | - |
| Mitoku et al. 2016 | 1754 | 320 | 81.8 (7.2) | 605 | 1149 | Japanese | Community | Baseline Cognitively Normal | - |
| Oh et al. 2023 | 55800 | 459 | 73.4 (5.8) | 24888 | 30912 | South Koreans | Community | Baseline Cognitively Normal | - |
| Pabst et al. 2021 | 3497 | 229 | 79.8 (3.9) | - | - | Germans | Community | Baseline Cognitively Normal | 27.5 (1.9) |
| Parada et al. 2021 | 1383 | 251 | 74.4 (9.4) | 541 | 842 | Americans (Predominantly White) | Community | Both Baseline Cognitive Impairment and Baseline Cognitively Normal | - |
| Schneck et al. 2012 | 446 | 10 | 79.9 (7.5) | 192 | 254 | Americans | Community | - | - |
| Schneider et al. 2012 | 1972 | 119 | - | 840 | 1132 | Australians | Community | - | - |
| Tomida et al. 2022 | 4471 | 510 | 75.9 (4.2) | 2131 | 2340 | Japanese | Community | Both Baseline Cognitive Impairment and Baseline Cognitively Normal | - |
| Wong et al. 2023 | 2574641 | 3055 | - | 1098663 | 1475976 | Americans - White 1659272, Black 110821, Asian 321261, Hispanic 399043, Other or Unknown: 84244 | Community | - | - |
| Yamada et al. 2016 | 1989 | 122 | 83.3 (IQR: 11.1) | 616 | 1373 | Czechs, English, Finnish, French, Germans, Israelis, Italians, Dutch | Institutionalised | Both Baseline Cognitive Impairment and Baseline Cognitively Normal | 1.9 (1.2) |

Supplemental Table S4**: Evaluation of Risk of Bias Using The ROBINS-E Scale for Studies Investigating The Global Prevalence of DSI.**


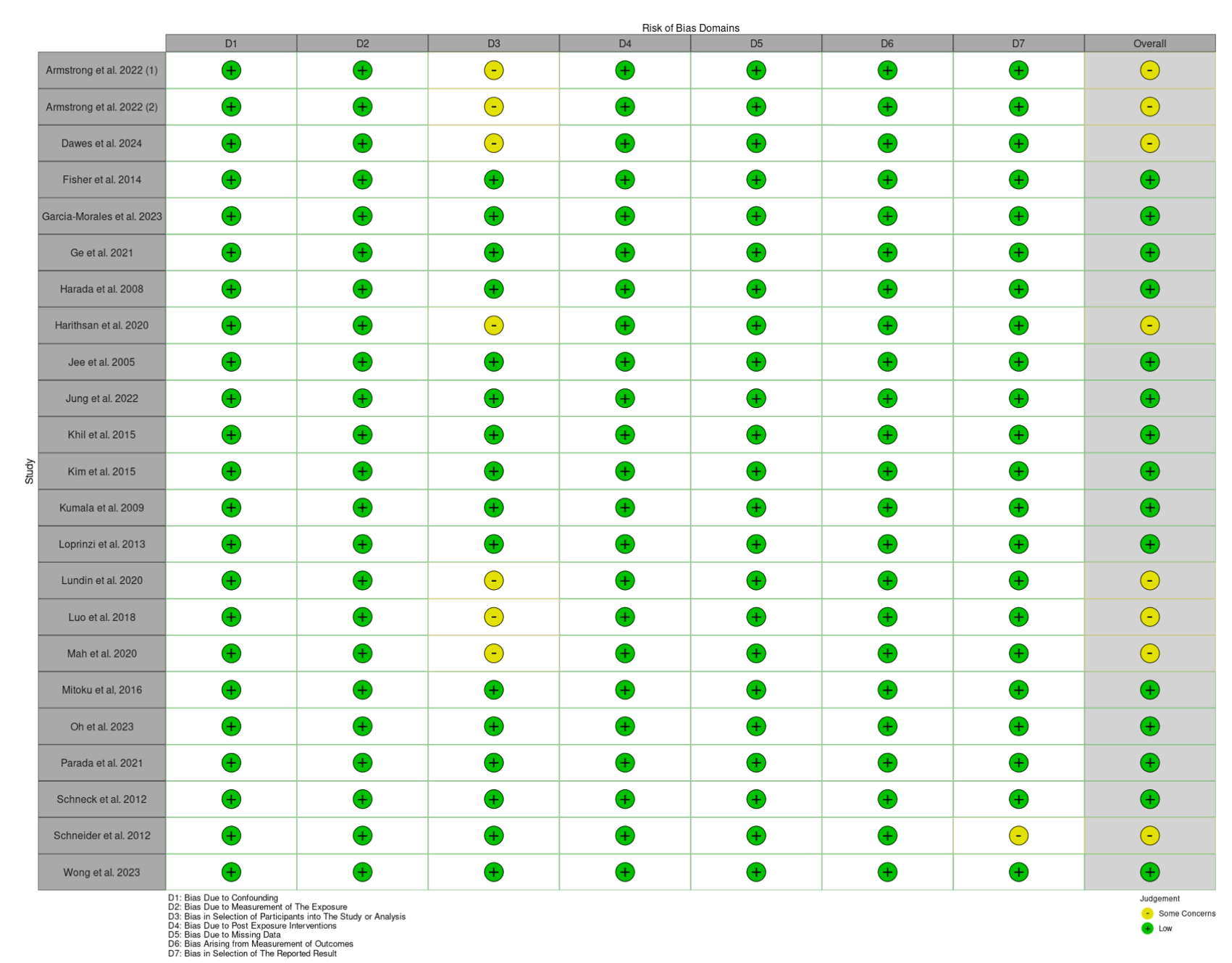


Supplemental Table S5**: Evaluation of Risk of Bias Using The ROBINS-E Scale for Studies Investigating The Association of DSI and Cognition.**


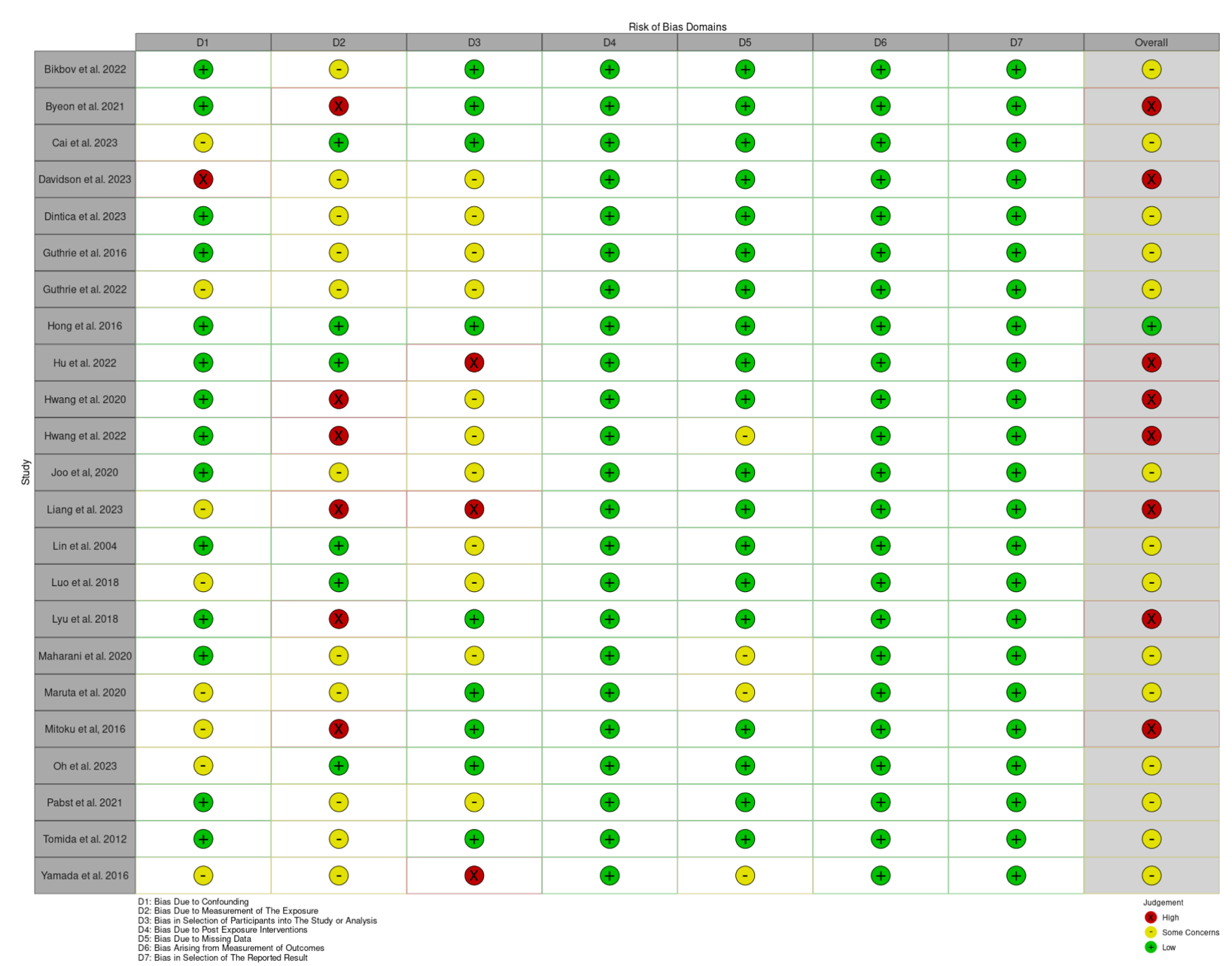


Supplemental Table S6**: Trim-And-Fill Sensitivity Analyses.**

| **Outcome** | **Original Meta-Analysis** | | | **Egger’s Test** | |
| --- | --- | --- | --- | --- | --- |
|  | **Studies** | **Estimate (95% CI)** | **I^2^ (%)** | **Intercept** | **P-value** |
| Global Prevalence of DSI (%) | 23 | 5.50 (2.88-10.26) | 100 | 22.012 | 0.062 |
| Association of DSI with Prevalent Cognitive Impairment (OR) | 11 | 1.71 (1.36-2.16) | 99.7 | -0.891 | 0.898 |
| Association of DSI with Incident Cognitive Impairment (OR) | 16 | 1.72 (1.37-2.15) | 91.8 | -1.005 | 0.524 |

Abbreviations: DSI: Dual Sensory Impairment; OR: Odds Ratio; CI: Confidence Interval.

Supplemental Table S7**: Random-Effects Meta-Regression of Log(Prevalence) or Log(OR) Against Potential Effect Moderators (Continuous and Categorical Characteristics).**

| **Analysis** | **Covariate** | **Beta‡** | **SE** | **Z** | **P** | **95% CI Lower** | **95% CI Upper** | **I^2^ (% Residual Heterogeneity)** | **R^2^ (% Accounted Heterogeneity)** |
| --- | --- | --- | --- | --- | --- | --- | --- | --- | --- |
| Global Prevalence of DSI | Age Group (60-69, 70-79, 80+) | 0.1118 | 0.0513 | 2.1778 | **0.0294*** | 0.0112 | 0.2123 | 99.86 | 13.03 |
|  | % Male | -0.0204 | 0.0347 | -0.5864 | 0.5576 | -0.0885 | 0.0477 | 99.95 | - |
|  | Study Design (Cross-Sectional vs. Cohort) | -1.3840 | 0.6867 | -2.0155 | 0.0439^ | -2.7299 | -0.0381 | 99.94 | - |
| Baseline DSI and Incident Cognitive Decline | Average Age (Years) | 0.0032 | 0.0066 | 0.4840 | 0.6284 | -0.0097 | 0.0161 | 38.97 | - |
|  | Study Design (Cross-Sectional vs. Others) | 0.1341 | 0.2370 | 0.5658 | 0.5715 | -0.3304 | 0.5986 | 92.06 | - |
|  | Follow-Up Duration (Years) | 0.0033 | 0.0035 | 0.9402 | 0.3471 | -0.0035 | 0.0100 | 91.59 | - |
|  | Method of Sensory Assessment (subjective vs. Objective) | 0.1754 | 0.2583 | 0.6791 | 0.4971 | -0.3309 | 0.6818 | 92.08 | - |

Abbreviations: DSI: Dual Sensory Impairment; OR: Odds Ratio; CI: Confidence Interval.

*Age Group: P-value for permutation test with 1,000 iterations = 0.0420, which suggests true effect moderation.

^Study Design: P-value for permutation test with 1,000 iterations = 0.0650, which suggests spurious effect moderation.
‡ Estimated factor by which the log(prevalence) or log(OR) changes per unit increase in a continuous variable or in comparison with the reference group for a categorical variable. 95% CIs are also presented in log scale.

Supplemental Table S8**: Population Attributable Fraction of Incident Cognitive Decline Associated With Dual Sensory Impairment.**

|  | **Age 18+** | | | **Age 60-69** | | | **Age 70-79** | | | **Age 80+** | | |
| --- | --- | --- | --- | --- | --- | --- | --- | --- | --- | --- | --- | --- |
|  | **PAF (%)** | Lower CI | Upper CI | **PAF (%)** | Lower CI | Upper CI | **PAF (%)** | Lower CI | Upper CI | **PAF (%)** | Lower CI | Upper CI |
| World | **3.41** | 0.91 | 9.85 | **1.17** | 0.14 | 7.32 | **3.67** | 0.53 | 16.78 | **9.20** | 1.49 | 30.74 |
| Asia | **2.89** | 0.66 | 9.62 | **3.26** | 1.22 | 6.96 | **7.49** | 2.39 | 17.52 | **15.94** | 7.22 | 27.18 |
| North America | **4.92** | 0.71 | 21.10 | **0.31** | 0.01 | 9.06 | **3.62** | 0.27 | 25.09 | **10.27** | 0.77 | 41.83 |
| Europe | **1.64** | 0.19 | 10.27 |  |  |  |  |  |  |  |  |  |
| Oceania | **7.36** | 1.54 | 22.91 |  |  |  |  |  |  |  |  |  |

Abbreviations: PAF: Population Attributable Fraction (%); DSI: Dual Sensory Impairment; CI: Confidence Interval.

Supplemental Table S9**: Population Attributable Fraction of Incident Dementia Associated With Dual Sensory Impairment.**

|  | **Age 18+** | | | **Age 60-69** | | | **Age 70-79** | | | **Age 80+** | | |
| --- | --- | --- | --- | --- | --- | --- | --- | --- | --- | --- | --- | --- |
|  | **PAF (%)** | Lower CI | Upper CI | **PAF (%)** | Lower CI | Upper CI | **PAF (%)** | Lower CI | Upper CI | **PAF (%)** | Lower CI | Upper CI |
| World | **2.78** | 0.94 | 7.15 | **0.85** | 0.12 | 4.90 | **2.68** | 0.47 | 11.62 | **6.82** | 1.33 | 22.45 |
| Asia | **2.35** | 0.71 | 6.69 | **2.37** | 1.09 | 4.65 | **5.52** | 2.14 | 12.17 | **12.05** | 6.49 | 19.58 |
| North America | **4.28** | 0.84 | 15.80 | **0.23** | 0.01 | 6.10 | **2.64** | 0.24 | 17.93 | **7.64** | 0.69 | 31.93 |
| Europe | **1.19** | 0.17 | 6.94 |  |  |  |  |  |  |  |  |  |
| Oceania | **5.42** | 1.38 | 16.23 |  |  |  |  |  |  |  |  |  |

Abbreviations: PAF: Population Attributable Fraction (%); DSI: Dual Sensory Impairment; CI: Confidence Interval.

Supplemental Table S10**: Evaluation of Quality Of Pooled Evidence Using The Grading of Recommendations Assessment, Development and Evaluation (GRADE) Framework.**

| **Number of Studies** | **Certainty Assessment** | | | | | | **Effect** | | | **Certainty** |
| --- | --- | --- | --- | --- | --- | --- | --- | --- | --- | --- |
|  | **Study Design** | **Risk of Bias** | **Inconsistency** | **Indirectness** | **Imprecision** | **Other Considerations** | **Events** | **Individuals** | **Rate/Ratio (95% CI)** |  |
| Global Pooled Prevalence of DSI | | | | | | | | | | |
| 23 | Non-Randomised Studies | Not Serious | Serious | Not Serious | Not Serious | None | 215 | 3920929 | Event Rate 5.50 per 100 (2.88 to 10.26) | ⨁⨁⨁◯ Moderate |
| Global Pooled Prevalence of Dementia Among Patients with DSI | | | | | | | | | | |
| 3 | Non-Randomised Studies | Not Serious | Serious | Not Serious | Not Serious | None | 18563 | 144943 | Event Rate 5.20 per 100 (1.63 to 15.34) | ⨁⨁⨁◯ Moderate |
| Global Pooled Prevalence of Cognitive Impairment Among Patients With DSI | | | | | | | | | | |
| 5 | Non-Randomised Studies | Not Serious | Very Serious | Not Serious | Not Serious | None | 114370 | 138041 | Event Rate 59.83 per 100 (41.03 to 76.12) | ⨁⨁◯◯ Low |
| Association of DSI With Prevalent Cognitive Impairment and Dementia | | | | | | | | | | |
| 11 | Non-Randomised Studies | Not Serious | Not Serious | Not Serious | Not Serious | All Plausible Residual Confounding Would Reduce The Demonstrated Effect |  |  | Odds Ratio 1.71 (1.35 to 2.16) | ⨁⨁⨁◯ Moderate |
| Association of DSI with Incident Cognitive Decline | | | | | | | | | | |
| 16 | Non-Randomised Studies | Not Serious | Not Serious | Not Serious | Not Serious | All Plausible Residual Confounding Would Reduce The Demonstrated Effect |  |  | Odds Ratio 1.72 (1.27 to 2.15) | ⨁⨁⨁◯ Moderate |

Abbreviations: DSI: Dual Sensory Impairment
